# Organic-chemical fertilizer combination enhances maize yield through coordinated regulation of leaf C-N metabolism, photosynthesis and dry matter partitioning in northeast black soil, China

**DOI:** 10.3389/fpls.2026.1879928

**Published:** 2026-07-02

**Authors:** Meng Cheng, Rong Jiang, Yanhua Chen, Yubo Hao, Yang Yu, Guoyi Lv, Yiteng Zhang, Yubo Jiang, Runze Wang, Ziwen Liu, Jingwen Wei, Chunrong Qian, Guoyuan Zou, Wanrong Gu

**Affiliations:** 1 College of Agriculture, Northeast Agricultural University, Harbin, China; 2 The Institute of Plant Nutrition, Resources and Environment, Beijing Academy of Agriculture and Forestry Sciences, Beijing, China; 3 Institute of Crop Cultivation and Farming, Heilongjiang Academy of Agricultural Sciences, Harbin, China

**Keywords:** dry matter accumulation, leaf senescence, nitrogen and carbon metabolism, organic-chemical fertilization, photosynthetic performance

## Abstract

Aiming at how organic fertilizer regulates the growth and development of maize in the Northeast black soil region, and to explore sustainable nutrient management models, this study aims to investigate how it regulates the photosynthetic physiological characteristics, leaf senescence dynamics, dry matter accumulation and translocation, grain filling characteristics, and final yield formation of maize. A long-term field trial was conducted using maize cultivar Jingnongke 728, with five treatments: conventional fertilization (CK), full substitution of chemical fertilizer with organic fertilizer, and organic fertilizer substituting 10%, 20%, and 30% of chemical fertilizer. Results showed that the 10% organic substitution treatment significantly improved leaf photosynthetic capacity, including higher relative chlorophyll content (SPAD), actual PSII photochemical efficiency (*Y(II)*), photosynthetic characteristics, and activities of carbon-nitrogen metabolism enzymes (1,5-bisphosphate (RuBP) carboxylase, phosphoenolpyruvate carboxylase (PEPC), nitrate reductase (NR), glutamine synthetase (GS), glutamate oxoglutarate aminotransferase (GOGAT), glutamic oxaloacetic transaminase (GOT), glutamic pyruvic transaminase (GPT), glutamate dehydrogenase (GDH). The 20% organic substitution treatment achieved optimal performance at the silking (R1) and milk (R3) stages. In terms of leaf area, the 10% and 20% substitution treatments increased the maximum leaf area by 4.75%-22.22% (2024) and 2.10%-7.41% (2025) respectively, and all organic substitution treatments delayed the maximum leaf senescence rate. For dry matter accumulation, the 10% substitution promoted early vegetative growth, while the 20% substitution benefited late reproductive growth; both modulated dry matter contribution to grain formation and extended the active grain filling period (up to 51.65 days in 2024 and 52.19 days in 2025 for 20% substitution). Regarding yield, all organic-chemical combination treatments increased yield, with the 10% and 20% substitution treatments showing the best effects replacing 10% of chemical fertilizer with organic fertilizer increased yield by 3.58% in 2024 and 3.30% in 2025, while replacing 20% of chemical fertilizer with organic fertilizer increased yield by 1.87% in 2024 and 5.25% in 2025. Full organic substitution reduced kernels per ear and yield. In conclusion, organic fertilizer slows leaf senescence, but sole organic substitution impairs early nutrient uptake and yield. Substituting 10%-20% of chemical fertilizer with organic fertilizer balances early and late nutrient supply, enhances photosynthetic capacity

## 1 Introduction

Among cultivation practices in maize production, optimizing fertilizer management is a key measure for regulating maize grain filling and yield, while applying organic fertilizers serves as an important strategy for integrating traditional agriculture with modern green production (Jiang et al., 2022). In the black soil region of Northeast China, among animal manure and crop straw, livestock manure is mainly cattle manure, with a total output of 31.60×10^8^ t, accounting for 52.44%; and straw is mainly maize straw, with a total output of 18.40×10^8^ t, accounting for 63.30% (Tian, 2024). Utilizing localized agricultural waste resources not only reduces waste disposal costs and environmental pollution risks but also mitigates agricultural depletion of soil resources. The Northeast Black Soil Region possesses abundant organic fertilizer resources with significant potential. Organic components promote the proliferation of beneficial microorganisms (Köninger et al., 2021; Kim et al., 2022). The metabolic activities of these microorganisms further enrich soil organic matter and enhance nutrient cycling. However, although organic fertilizer is rich in organic matter and nutrients, has a long-lasting fertilizer effect, and can achieve soil fertility improvement, it has low nutrient content and a slow release rate (Hong et al., 2017). Due to their slow-release nature, sole application often leads to reduced crop dry matter accumulation and yield losses (Yu et al., 2021; Wei et al., 2023). Chemical fertilizers, in contrast, exhibit short-term nutrient effects but offer high nutrient content, rapid release, and strong yield-enhancing effects (Cai et al., 2015). The combined application of organic and inorganic fertilizers profoundly alters the balance of soil nitrogen mineralization and immobilization by regulating the soil carbon-to-nitrogen ratio, microbial activity, and nutrient adsorption capacity. Research has shown that, compared to urea application alone, the co-application of urea with rice straw biochar or poultry manure biochar reduces the nitrogen mineralization rate by 19%-28% and 38%-45%, respectively (Dey and Mavi, 2021). The combined application of organic and inorganic fertilizers can optimize soil nitrogen supply and crop nitrogen uptake both temporally and spatially (Huo et al., 2015), reducing resource waste and environmental pollution while decreasing the agricultural production system’s dependence on chemical fertilizers.

However, the underlying mechanisms by which different substitution ratios precisely regulate the entire chain from photosynthetic physiology to yield formation still require systematic investigation (Wang et al., 2024). The effect of organic fertilizer depends not only on the substitution ratio but also on its source, degree of maturity, and soil environment. Studies have shown that the application of insect frass organic fertilizer derived from black soldier fly larvae composting of kitchen waste significantly increases the net photosynthetic rate and root activity of maize seedlings (Wu et al., 2023). Under drought stress, the combined application of a trichoderma-containing bio-inoculant and organic fertilizer significantly alleviates the inhibition of maize CO_2_ assimilation rate and maintains a higher photosynthetic efficiency (Radzikowska-Kujawska et al., 2025). Thus, the regulation of photosynthetic physiology by combined organic and inorganic fertilizer application is a complex process involving multiple interacting factors.

From the perspective of leaf photosynthetic capacity, 1,5-bisphosphate (RuBP) carboxylase and phosphoenolpyruvate carboxylase (PEPC) are key enzymes in CO_2_ fixation during photosynthesis and determine a plant’s photosynthetic capacity nitrate reductase (NR), glutamine synthetase (GS), and glutamate oxoglutarate aminotransferase (GOGAT) are three crucial enzymes in the nitrogen metabolism pathway, collectively facilitating the conversion of absorbed nitrate into organic nitrogen. Glutamate oxidase (GOT), glutamic pyruvic transaminase (GPT), and glutamate dehydrogenase (GDH) participate in the synthesis and breakdown of amino acids, as well as the redistribution of carbon and nitrogen skeletons. Nitrogen is a key structural and functional component of these enzymes, directly determining the capacity for light harvesting and carbon-nitrogen metabolism (Fan et al., 2026). Similarly, a highly photosynthetic C4 crop, optimizing maize growth phenotypes not only maintains a high leaf area index but also enhances net photosynthetic rate and light use efficiency, thereby fully realizing yield potential (Yue et al., 2022). Adequate nitrogen supply not only promotes dry matter accumulation during the early to mid-grain filling stages and increases aboveground biomass (Guo et al., 2025), but also maintains high photosynthetic capacity during grain filling to enhance photosynthetic efficiency (Liu et al., 2019).

Ultimately, the optimization of photosynthetic physiology and the delay in leaf senescence jointly affect dry matter production, distribution, and transport, thereby influencing yield components (He et al., 2019; Li et al., 2022; Yan et al., 2023). Studies have shown that an appropriate organic fertilizer substitution ratio increases the kernel number per ear and 100-kernel weight of maize, thus achieving higher yields (Wang et al., 2024). However, the overall effect of organic fertilizer application remains lower than that of inorganic inputs (Kadigi et al., 2025). Research indicates that maize photosynthetic performance and growth parameters first increase and then decrease with increasing organic fertilizer substitution ratios, peaking at a substitution ratio of 20% (Han et al., 2025). Excessively high substitution ratios may inhibit carbon and nitrogen metabolism due to insufficient initial nutrient supply, leading to reduced dry matter accumulation and yield. A meta-analysis study showed that when the substitution ratio of organic fertilizer for chemical fertilizer exceeded 70%, vegetable yield decreased by 13.6% (Liu et al., 2021b). During organic fertilizer application, the ratio of organic to chemical fertilizer is a key factor determining crop growth and development. Therefore, exploring the optimal organic fertilizer substitution ratio is crucial for balancing yield and environmental goals (Wang et al., 2025). There is still room for exploration regarding the ratio of organic to chemical fertilizer, and further investigation is needed on how nutrient regulation can modify plant growth and development.

Therefore, this paper will investigate the relationship between maize growth dynamics, nutrient demand, and yield formation under different combined application schemes from the perspectives of leaf carbon and nitrogen metabolism, photosynthetic capacity, dry matter accumulation, distribution and transport, post−flowering leaf senescence, and grain filling characteristics throughout the whole growth cycle, in order to explore the effects of different organic fertilizer substitution ratios on maize yield and yield components.

## 2 Methods

### 2.1 Site description

The field trial was conducted at the Modern Agricultural Demonstration Zone of the Heilongjiang Academy of Agricultural Sciences in Harbin City, Heilongjiang Province, China (45°50’54’’N, 126°50’12’’E, altitude 128 m). The experimental area belongs to the first accumulated temperature zone of Heilongjiang Province, with active accumulated temperature (≥ 10 °C) exceeding 2700 °C and an annual frost-free period of 135–145 days. The annual mean temperature ranges from 3.5 to 4.5 °C, with annual precipitation between 400 and 600 mm. Total precipitation from April to September in 2024 and 2025 was 591.3 mm and 414.1 mm, respectively, with sunshine hours of 1297.9 h and 1612.4 h (**Supplementary Figure 1**). The soil type in the experimental area is meadow soil. The basic nutrient characteristics of the 0–20 cm soil layer in the experimental area are as follows: soil organic carbon (SOC) content is 16.24 g/kg, soil total nitrogen (TN) content is 1.74 g/kg, total phosphorus (TP) content is 0.49 g/kg, total potassium (TK) content is 15.5 g/kg, available nitrogen (AN) content is 136.66 mg/kg, available phosphorus (AP) content is 27.6 mg/kg, available potassium (AK) content is 134.2 mg/kg, and soil pH is 6.52.

### 2.2 Experimental materials

Test variety Jingnongke 728 exhibits excellent quality, tolerance to dense planting, lodging resistance, good overall disease resistance, stable yield potential, rapid desiccation, early maturity, and suitability for mechanical harvesting. It is recommended for cultivation in regions with effective accumulated temperatures of approximately 2500 °C (Li et al., 2019).

### 2.3 Experimental design

The experiment was based on the 2019 long-term fixed-point trial, with the following treatments designed: conventional fertilization CK (600 kg/ha chemical fertilizer). Organic fertilizer replacing 100% chemical fertilizer T1 (15,000 kg/ha organic fertilizer). Organic fertilizer replacing 10% chemical fertilizer T2 (7,500 kg/ha organic fertilizer, 540 kg/ha chemical fertilizer). Organic fertilizer replacing 20% chemical fertilizer T3 (7,500 kg/ha organic fertilizer, 480 kg/ha chemical fertilizer). Organic fertilizer replacing 30% chemical fertilizer T4 (7500 kg/ha organic fertilizer, 420 kg/ha chemical fertilizer). Each plot consists of 6 rows with a row length of 39 m. The sowing density is 67,500 plants per hectare, with a plant spacing of 0.228 m and a row spacing of 0.65 m. In autumn, all chemical fertilizer (Anhui Maoshi New Fertilizer Co., Ltd., N-P_2_O_5_-K_2_O=29-10-13) and organic fertilizer (Heilongjiang Lianzeng Agriculture and Animal Husbandry Technology Co., Ltd.) were applied as base fertilizer in one application during soil preparation. The organic fertilizer is produced by the decomposition of crop straw and contains the following basic nutrients: organic matter 120.07 g/kg, N 18.7 g/kg, P 14.78 g/kg, and K 23.27 g/kg. Field management was the same as conventional production. This experiment was conducted under rain-fed conditions, with no artificial irrigation throughout the entire growth period. In the experimental plots, maize was planted on April 27, 2024, and harvested on September 28; in 2025, it was planted on April 28 and harvested on September 26 for yield measurement.

### 2.4 Sampling and measurements

#### 2.4.1 Leaf chlorophyll relative content (SPAD)

A portable chlorophyll meter model SPAD-502 Plus (Konica Minolta, Tokyo, Japan) was used to measure maize leaves at four growth stages: V6 (Sixth Leaf Stage), V12 (Twelfth Leaf Stage), R1 (Silking Stage), and R3 (Milk Stage). Measurements were taken on clear, cloudless days between 10:00 and 14:00. Ten representative plants were selected from each plot. At the V6 stage, the sixth expanded leaf was chosen, at V12, the twelfth expanded leaf. At R1 and R3, the ear position leaf was selected. The SPAD value was measured and recorded at the right side of the midrib in the leaf mid-section.

#### 2.4.2 Leaf chlorophyll fluorescence parameters

Chlorophyll fluorescence parameters were measured at maize growth stages V6, V12, R1, and R3 using the MINI-PAM-II ultra-portable handheld chlorophyll fluorometer (WALZ GmbH, Nuremberg, Germany). Measurements were conducted on clear days between 9:00 and 11:00 AM. Three representative plants per row were selected for each treatment. Using the instrument’s leaf clamp, leaves (selected as described above) were dark-treated for 30 minutes at the midpoint, avoiding the leaf veins. Calculate PSII maximum photochemical efficiency (*F_v_/F_m_*), PSII actual photochemical efficiency *Y(II)*, and non-photochemical quenching coefficient (*NPQ*) (Li et al., 2025b).

#### 2.4.3 Leaf photosynthetic characteristics

A portable photosynthesis meter (LI-6400XT, LI-COR, USA) was used to measure maize leaves at the V6, V12, R1, and R3 stages. Measurements were taken on clear, cloudless days between 9:00 and 11:00 am using the built-in light source, with natural light intensity set to 1600 μmol·m^-2^·s^-1^. Ten representative plants with uniform light exposure were selected from each plot. At the V12 stage, the twelfth expanded leaf was chosen. At the R1 and R3 stages, the ear-position leaf was selected. Measurements of net photosynthetic rate (*P_n_*), transpiration rate (*T_r_*), stomatal conductance (*G_s_*), and intercellular CO_2_ concentration (*C_i_*) were taken at the mid-vein right side of the leaf.

#### 2.4.4 Activity of key enzymes in leaf carbon and nitrogen metabolism

At stages V12, R1, and R3, select three plants per treatment with uniform growth and consistent light exposure to collect functional leaves (leaf selection method as above). The mid-vein section was excised from the leaf center, rapidly frozen in liquid nitrogen, and transported to the laboratory. Samples were stored at -80 °C in an ultra-low temperature freezer for subsequent analysis. Enzyme activities of 1,5-bisphosphate (RuBP) carboxylase, and phosphoenolpyruvate carboxylase (PEPC), both key enzymes in carbon metabolism, were measured. Nitrate reductase (NR), glutamine synthetase (GS), and glutamate oxoglutarate aminotransferase (GOGAT) (Singh and Srivastava, 1986) associated with nitrogen metabolism, glutamic oxaloacetic transaminase (GOT), glutamic pyruvic transaminase (GPT) (Wu et al., 1998), and glutamate dehydrogenase (GDH) activity (Gonzalez et al., 1993).

#### 2.4.5 Leaf senescence process

##### 2.4.5.1 Green leaf area index

After leaf area reaches its maximum during the tasseling stage (VT) of maize, select three representative plants with uniform growth from each plot. Remove all green leaves from these plants and measure the total green leaf area per individual plant using the length-width coefficient method (Qian et al., 2016). The calculation formula is as follows:


$$LA=0.75\times \sum_{i=1}^{n}(L\times W)$$

In the formula, LA is measured in m^2^, 0.75 is the correction factor, L and W are the maximum length and width of the blades, and n is the number of blades.

Select two rows of plants with no missing seedlings in the community to count the number of plants, convert their actual density into the actual number of plants in each community, and calculate the green leaf area index (Guo et al., 2022) (LAI). The calculation formula is as follows:


$$LAI=\frac{LA\times P}{GA}$$

In the formula, P represents the actual number of plants in each community, and GA represents the land area of each community in m^2^.

##### 2.4.5.2 Leaf senescence fitting

When maize enters the R1 stage, record the silk emergence time of maize and record this period as the starting time of leaf aging. From then on, record the degree of leaf aging of maize every seven days, and record that more than half of the leaves are withered or yellow as aging until the seeds are ripe and harvested. Fit the dynamic changes of green leaf area index throughout the entire aging cycle using a quadratic equation, and observe the aging differences of maize leaves under different treatments through the fitting model. Fit the dynamic changes in the green area of maize leaves into a logistic equation (Lan et al., 2024):


$$y=\frac{a}{1+e^{-b(x-c)}}$$

Where x is the number of days after silk emergence (d), y is LAI, a is the theoretical initial value of LA, b is a constant describing the aging rate, and c is the time to reach the maximum aging rate.

Then take the first derivative to calculate the rate of change, and take the second derivative to find the maximum rate inflection point:


$$\frac{dy}{dx}=a\times \frac{b\times e^{-b(x-c)}}{{\left(1+e^{-b(x-c)}\right)}^{2}}$$

The point where the second derivative equals zero is the inflection point ( $x=x_{c}$). When ( $x=x_{c}$), $e^{-k(x-x_{c})}=e^{0}=1$, substitute into the first derivative to obtain the maximum rate.


$$V_{max}=\frac{dy}{dx}{|}_{x=c}\;=a\times \frac{b\times 1}{{\left(1+1\right)}^{2}}=\frac{a\times b}{4}$$

#### 2.4.6 Grain filling characteristics

Mark plants with consistent silk emergence during the R1 stage. Begin sampling 7 days after silk emergence, selecting 3 plants per treatment. Remove 100 kernels from the middle section of each ear, dry them in an 80 °C oven to constant weight, weigh the dry mass, and simulate the grain filling process using a logistic equation.


$$y=\frac{a}{1+be^{-kx}}$$

In the equation, a represents the final dry matter growth of the grain, b denotes the initial parameter, and k signifies the growth rate parameter. By simulating the grain filling process, the following filling characteristic parameters were obtained (Liang et al., 2025):


$$T_{max}=\frac{\ln b}{k}$$

$$W_{max}=\frac{a}{2}$$

$$R_{max}=\frac{\left(kW_{max}\right)}{\left(1-W_{max}/a\right)}$$

$$P=\frac{6}{k}$$

T_max_ indicates the time required to reach the maximum grain filling rate (d), W_max_ denotes the 100- grain weight at the maximum grain filling rate (g), R_max_ represents the maximum grain filling rate (g/d) and P indicates the active grain filling period (d).

#### 2.4.7 Accumulation, distribution, and transportation of dry matter

The critical growth stages for maize selection are V6, V12, R1, R3, and R6. From each plot, select the aboveground parts of three representative plants with uniform growth. Place the collected samples in an oven at 105 °C for 30 minutes to kill green, then reduce the temperature to 80 °C and dry until constant weight is achieved. Allow samples to cool to room temperature before weighing them on a 0.001% precision balance.

During the R1 and R6 stages of maize development, select three representative plants with uniform growth from each plot and classify their aboveground parts. At R1 stage, the plant was divided into three parts: leaves, stem sheath (stem + leaf sheath), and female ears. At R6 stage, the plant was divided into four parts: leaves, stem sheath (stem + leaf sheath), bracts (bracts + cob axis), and grains. Sample processing followed the same procedure as above. The contribution rate of dry matter transport in nutritional organs during flowering to grain dry matter accumulation was calculated (Lu et al., 2024; Jia et al., 2026), using the following formula:


$$DMTA(g/plant)=W_{R1}-W_{R6}$$

In the equation, DMTA is the dry matter translocation amount, W_R1_ is the dry matter mass of nutritional organs in R1, W_R6_ is the dry matter mass of nutritional organs in R6.


$$DMTE(\%)=\frac{DMTA}{W_{R1}}\times 100$$

In the equation, DMTE is the dry matter translocation efficiency.


$$CRDMG(g/plant)=\frac{W_{transferred}}{W_{grain}}\times 100$$

In the equation, CRDMG is the contribution rate of dry matter to grains, W_transferred_ is the dry matter mass transported from nutrient organs to grains, W_grain_ is the dry weight of the grain.

#### 2.4.8 Maize yield and its components

At the R6 stage, an area with uniform growth in the middle part of each plot was selected, with a measurement range of 8 m in length and 0.65 m in width. All ears within this area were collected for yield determination (converted to 14% standard moisture content), with five replicates per plot. Additionally, 25 representative ears were selected from each plot, air-dried, and subjected to seed examination. The number of rows per ear and kernels per row were recorded, the number of kernels per ear was calculated, and the 100-kernel weight and grain moisture content (using a grain moisture meter PM-8188-A, KETT, Japan) were measured. The yield (calculated at 14% moisture content) was then determined using the following formula:


$$Yield(t/ha)=\frac{W_{yield}\times (1-W_{moisture})}{(1-14\%)}\times \frac{10000}{M}$$

Where W_yield_ denotes the grain yield per plot (kg), W_moisture_ represents the grain moisture content per plot (%), and M indicates the sampling area (m²).

### 2.5 Data analysis

Data entry and calculations were performed using Microsoft Excel 365 software. One-way analysis of variance (ANOVA) was conducted using SPSS 26 software. Differences between treatments were analyzed for statistical significance at the *P* < 0.05 level using the LSD method. Graphs were generated using Origin 2024 software. All data in tables and figures are presented as mean ± standard deviation.

## 3 Results and analysis

### 3.1 Relative chlorophyll content (SPAD) and fluorescence parameters

**Figure 1:**
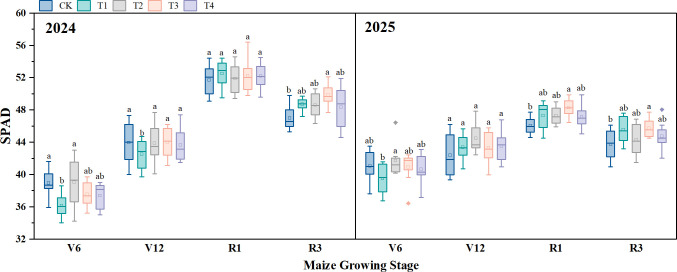
Differences in leaf SPAD values among treatments at different growth stages. Different letters indicate significant differences between different treatments (*P* < 0.05).

As shown in **Figure 1**, significant differences (*P* < 0.05) in leaf chlorophyll content were observed among different treatments over the two years. Overall, at the V6 stage, the trend was T2 > T3, T4, CK > T1; at the R3 stage, the trend was T3 > T1, T2, T1 > CK. During the V6 period, T2 increased by 0.16%-7.92% (2024) and 1.48%-5.33% (2025) compared to the other treatments. However, the differences between the two years fluctuated at the V12 and R1 stages. Among them, at the V12 stage, the T1 treatment decreased by 4.70%-5.37% compared to the other treatments; at the R1 stage in 2025, the T3 treatment increased by 4.61%, 1.95%, 1.98%, and 2.47% compared to CK, T1, T2, and T4, respectively. There were no significant differences in other periods.

**Figure 2:**
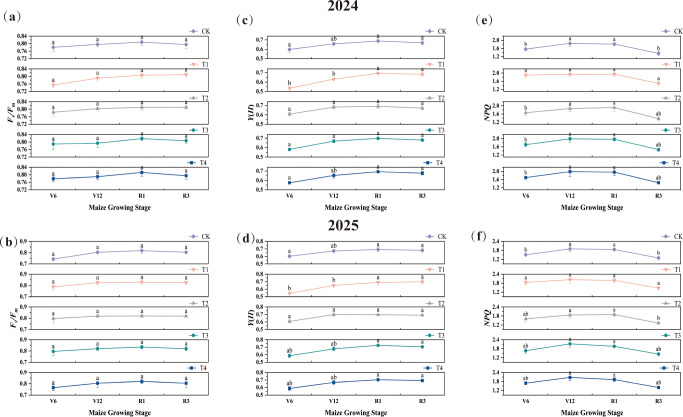
Differences in fluorescence parameters among treatments at different growth stages. Different letters indicate significant differences between different treatments (P < 0.05). In the image, **(a)** represents the maximum photochemical efficiency in 2024, **(b)** represents the maximum photochemical efficiency in 2025, **(c)** represents the actual photochemical efficiency in 2024, **(d)** represents the actual photochemical efficiency in 2025, **(e)** represents the non-photochemical quenching coefficient in 2024, and **(f)** represents the non-photochemical quenching coefficient in 2025.

Regarding fluorescence parameters (**Figure 2**), the trends in maximum photochemical efficiency (*F_v_/F_m_*) (**Figures 2a, b**) differed across periods during the two years but showed no significant differences (*P* < 0.05). Actual photochemical efficiency [*Y(II)*] (**Figures 2c, d**) showed significant differences between the V6 and V12 stages. During the V6 stage, the T1 treatment was significantly lower than the other treatments, with the T2 and T3 treatments in 2025 increasing by 3.45%-12.03% and 2.60%-11.11%, respectively, compared to the other treatments. During the V12 stage, the T2 treatment was significantly higher than the other treatments, increasing by 1.99%-8.08% (2024) and 2.95%-7.17% (2025), respectively, compared to the other treatments. Non-photochemical quenching (*NPQ*) coefficients (**Figures 2e, f**) showed significant differences between the V6 and R3 stages. During the V6 stage, the T1 treatment exhibited significantly higher *NPQ* values than other treatments, with increases ranging from 11.64% to 21.54% (2024) and 9.52% to 22.30% (2025). At the R3 stage, T1 showed the highest values while CK exhibited the lowest, with T1 increasing by 2.51%-10.21% (2024) and 2.65%-13.95% (2025) compared to other treatments. CK decreased by 1.09%-9.27% and 2.06%-12.24% relative to other treatments.

In terms of leaf SPAD and the fluorescence parameter *Y(II)*, at the key growth nodes of the early maize vegetative growth stage, V6 and V12, the T2 treatment generally showed the best increasing effect; whereas at the key growth nodes of the reproductive growth stage, R1 and R3, SPAD showed a more significant enhancement effect with T3, while among the five treatment groups, the differences in *Y(II)* were not significant. Treatment T1 without fertilizer application was significantly lower than other treatments at the early growth stages V6 and V12, but the gap narrowed at R1 and R3 after entering reproductive growth. In terms of *NPQ*, both at the V6 stage when maize just entered rapid growth and at the R3 stage when leaves began to senesce, the T2 treatment showed higher values, while at the R3 stage, the CK treatment was the lowest; no significant differences were observed in other stages.

### 3.2 Photosynthetic characteristics

**Figure 3:**
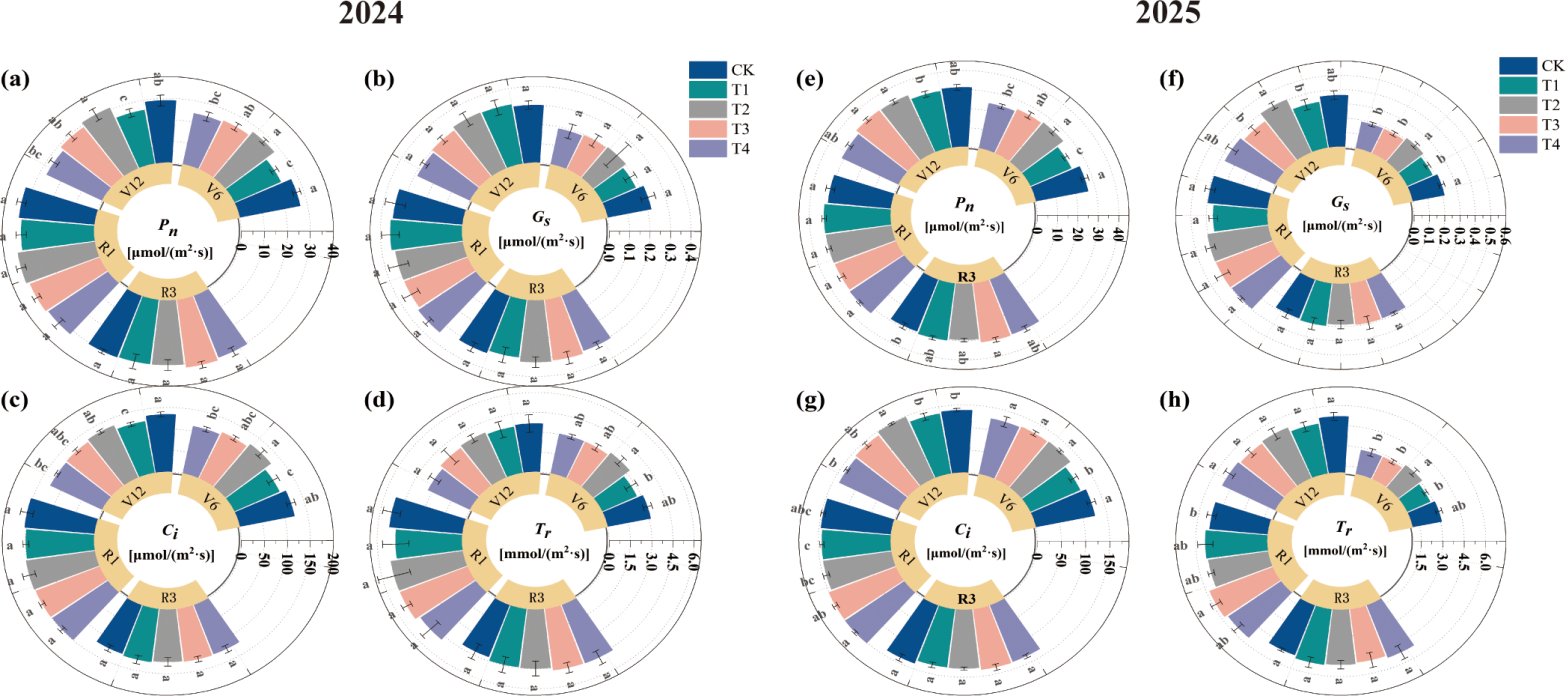
Differences in leaf photosynthetic characteristics among treatments at different growth stages. Different letters indicate significant differences between different treatments (P < 0.05). In the image, **(a)** represents the net photosynthetic rate in 2024, **(b)** represents the stomatal conductance in 2024, **(c)** represents the intercellular CO₂ concentration in 2024, **(d)** represents the transpiration rate in 2024, **(e)** represents the net photosynthetic rate in 2025, **(f)** represents the stomatal conductance in 2025, **(g)** represents the intercellular CO₂ concentration in 2025, and **(h)** represents the transpiration rate in 2025.

As shown in **Figure 3**, over the two years in terms of *P_n_* (**Figures 3a, e**), the T2 treatment showed significant differences (*P* < 0.05) at the V6 and V12 stages. At the V6 stage, T2 significantly increased by 1.19%-12.56% (2024) and 0.25%-18.22% (2025) compared with other treatments; at the V12 stage, it significantly increased by 6.03%-21.00% (2024) and 4.70%-8.10% (2025) compared with other treatments. The T3 treatment was significantly higher than other treatments at the V12 and R3 stages. At the V12 stage, the trend was T2, T3 > CK, T4 > T1, and it increased by 5.68%-9.12% compared with other treatments. At the R3 stage, the trend was T3 > T4, T1, T2 > CK, and it significantly increased by 6.94%, 3.40%, 5.80%, and 1.28% compared with CK, T1, T2, and T4, respectively. No significant differences were observed among treatments in other stages.

On stomatal conductance (*G_s_*), intercellular CO_2_ concentration (*C_i_*), and transpiration rate (*T_r_*), differences were more pronounced during the V6 and V12 stages, with the T2 treatment showing the most significant improvement. For *G_s_* (**Figures 3b, f**), no significant differences existed among treatments in 2024. In 2025, the T2 treatment increased yields by 12.27%-15.14% at V6 and 9.73%-23.91% at V12 compared to other treatments. For *C_i_* (**Figures 3c, g**), no significant differences were observed at the R3 stage. At other stages, T1 treatment exhibited lower values, reducing *C_i_* by 2.88%-14.76% (V6) and 2.94%-8.78% (V12) compared to other treatments in 2024. In 2025, reductions were 8.79%-11.91% (V6), 1.43%-8.88% (V12), and 0.34%-5.56% (R1). T2 treatment increased by 4.99%-17.32% (2024) in the V6 stage and 4.75%-9.74% (2025) in the V12 stage compared to other treatments. CK treatment increased by 1.04%-9.62% (2024) at the V12 stage, while T4 treatment increased by 0.24%-5.89% (2025) at the R1 stage. On *T_r_* (**Figures 3d, h**), during the V6 stage, the T2 treatment was significantly higher than other treatments in both years, increasing by 11.20%-17.89% (2024) and 2.06%-12.55% (2025) compared to other treatments. The T3 treatment was the highest in the R1 stage, with an increase of 4.29%-13.51% compared to other treatments (2025). There was no significant difference between the treatments in other stages.

Differences in photosynthetic characteristics among treatments were more pronounced during the V6 and V12 stages, with T2 treatment showing the greatest enhancement effect. However, the advantage of T2 diminished during the R1 and R3 stages after entering the reproductive phase, while R3 treatment gradually became more prominent across all groups. Similarly, the negative impact of T1 treatment without chemical fertilizer application on photosynthesis is more concentrated in the V6 and V12 stages.

### 3.3 Enzyme activity in leaves

#### 3.3.1 Enzyme activity related to carbon metabolism

**Figure 4:**
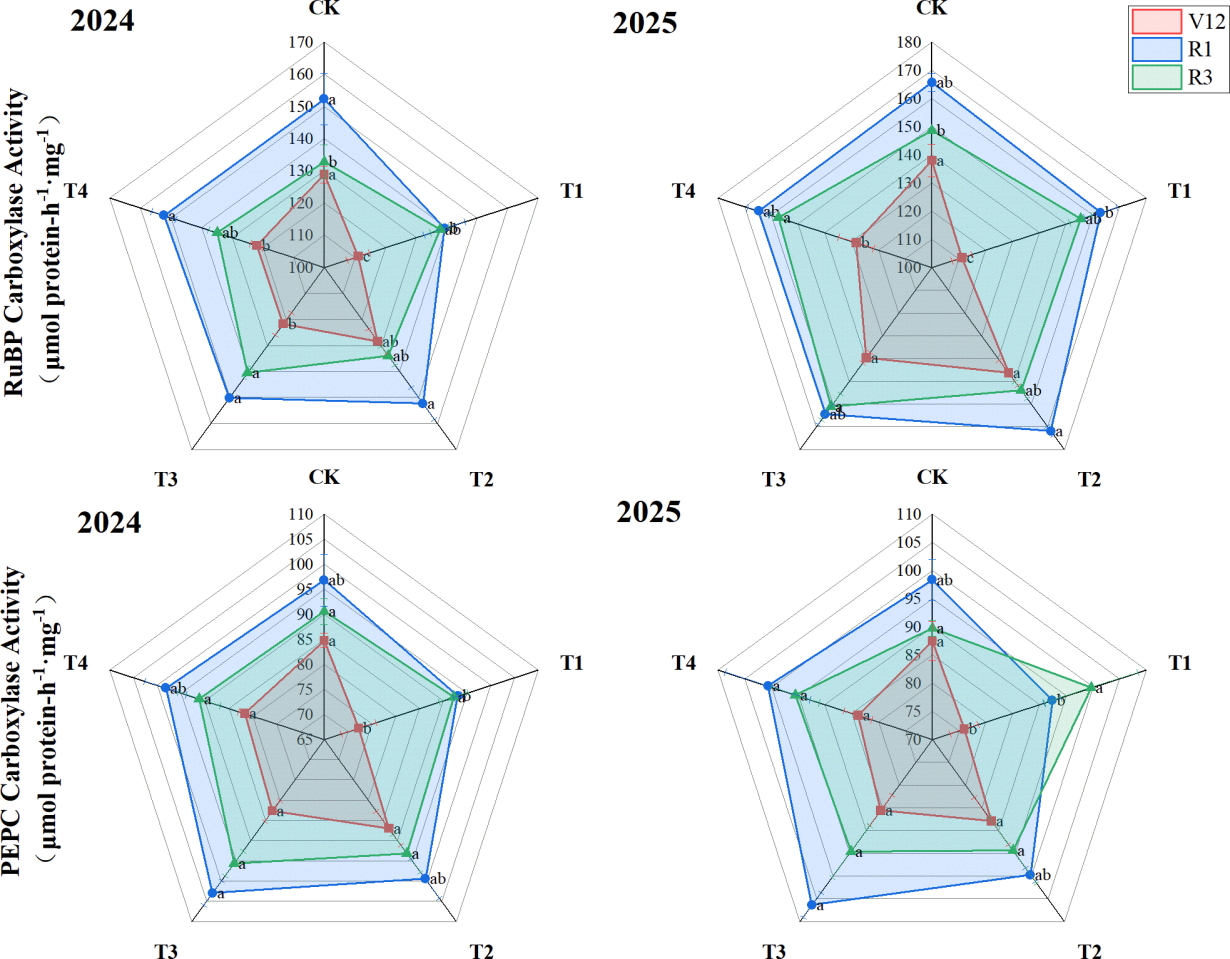
Differences in leaf carbon metabolism-related enzymes under different treatments at various growth stages. Different letters indicate significant differences between different treatments (P < 0.05).

As shown in **Figure 4**, significant differences (*P* < 0.05) were observed in the activities of 1,5-bisphosphate (RuBP) carboxylase and phosphoenolpyruvate carboxylase (PEPC) among different treatments at each growth stage. During the V12 and R1 stages, the T1 treatment exhibited significantly lower activity compared to other treatments. At the V12 and R1 stages, the T1 treatment was significantly lower than the other treatments. For RuBP carboxylase, at the V12 stage, the T1 treatment decreased by 8.61%-13.71% (2024) and 13.30%-23.99% (2025) compared to the other treatments, and at the R1 stage, it decreased by 7.17%-8.54% (2024) and 0.86%-5.10% (2025). For PEPC carboxylase, at the V12 stage, compared to the other treatments, it decreased by 11.56%-16.92% (2024) and 9.43%-13.59% (2025); at the R1 stage, it decreased by 3.87%-9.59% (2024) and 6.03%-13.11% (2025). T2 treatment increased RuBP carboxylase activity by 3.67%-5.37% during the R1 stage (2025). T3 treatment significantly enhanced RuBP carboxylase activity at the R3 stage, increasing it by 1.85%-5.71% (2024) and 2.49%-8.47% (2025), while also boosting PEPC activity at the R1 stage by 3.54%-10.61% (2024) and 5.63%-15.09% (2025).

In contrast, during the early rapid growth stage of maize (V12), the three treatments with the highest fertilizer application rates showed the best results. However, during the reproductive growth stages (R1 and R3), treatments combining organic and chemical fertilizers exhibited higher enzyme activity.

#### 3.3.2 Enzyme activity related to nitrogen metabolism

**Figure 5:**
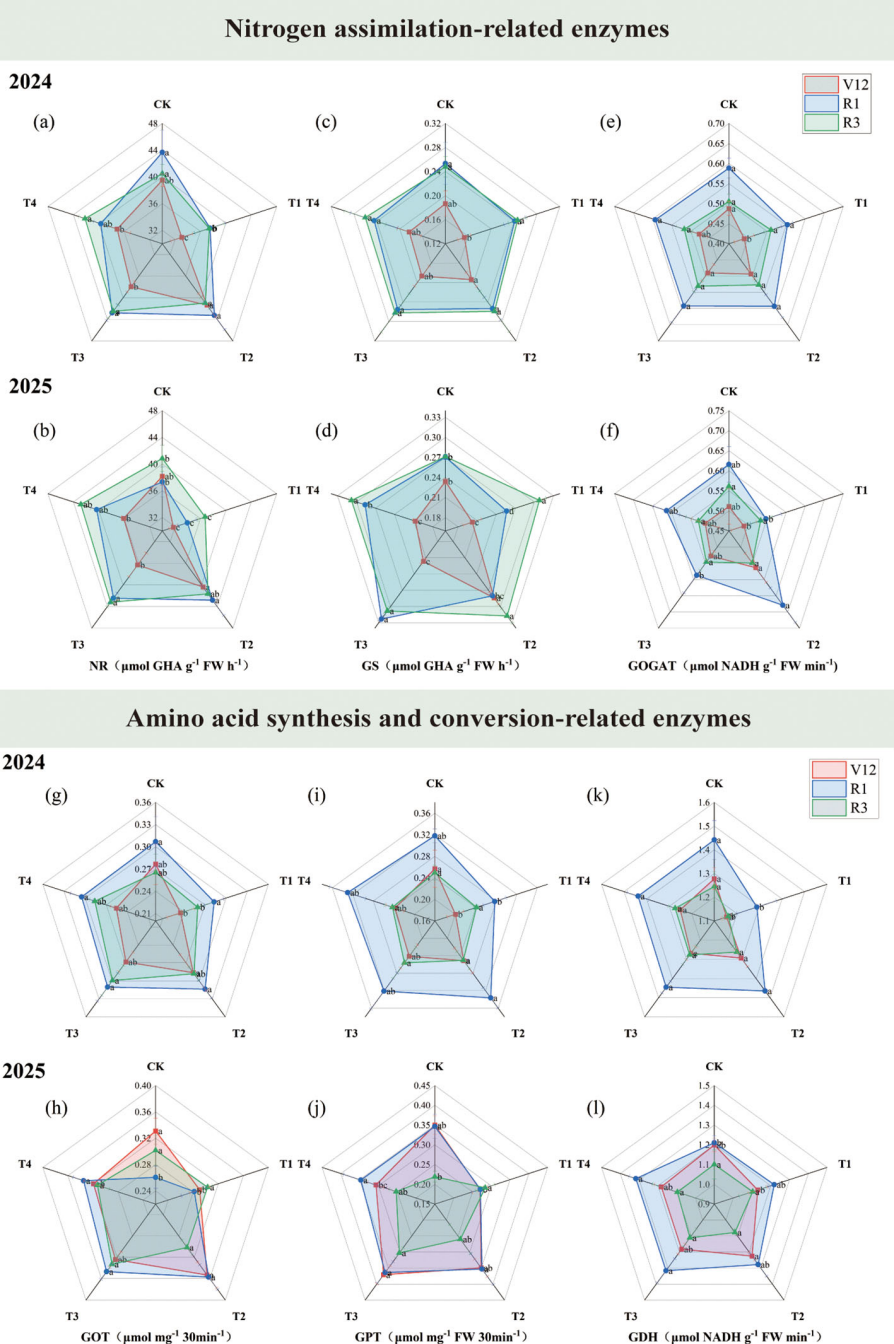
Differences of enzymes related to nitrogen assimilation and enzymes related to amino acid synthesis and transformation in leaves at different growth stages under different treatments. Different letters indicate significant differences between different treatments (P < 0.05). In the image, **(a)** represents the activity of nitrate reductase (NR) in 2024, **(b)** represents the activity of nitrate reductase (NR) in 2025, **(c)** represents the activity of glutamine synthetase (GS) in 2024, **(d)** represents the activity of glutamine synthetase (GS) in 2025, **(e)** represents the activity of glutamate oxoglutarate aminotransferase (GOGAT) in 2024, **(f)** represents the activity of glutamate oxoglutarate aminotransferase (GOGAT) in 2025, **(g)** represents the activity of glutamic-oxaloacetic transaminase (GOT) in 2024, **(h)** represents the activity of glutamic-oxaloacetic transaminase (GOT) in 2025, **(i)** represents the activity of glutamic-pyruvic transaminase (GPT) in 2024, **(j)** represents the activity of glutamic-pyruvic transaminase (GPT) in 2025, **(k)** represents the activity of glutamate dehydrogenase (GDH) in 2024, and **(l)** represents the activity of glutamate dehydrogenase (GDH) in 2025.

As shown in **Figure 5**, for nitrate reductase (NR) activity (**Figures 5a, b**), the T1 treatment exhibited lower activity than the other four treatments across all time points. The enzyme activity of the T2 treatment was higher than that of the other treatments at the V12 stage, increasing by 4.78%-25.14% (2024) and 5.82%-27.25% (2025), respectively, and at the R1 stage, it increased by 6.13%-26.08% (2025). Compared to the other treatments, the T3 treatment increased enzyme activity at the R1 stage by 5.24%-25.03% (2025), and at the R3 stage by 0.81%-17.52% (2025). Regarding glutamine synthetase (GS) activity (**Figures 5c, d**), at the V12 stage, the T1 treatment was significantly lower than other treatments, while the T2 treatment had the highest enzyme activity, increasing by 3.75%-26.35% (2024) and 21.37%-40.39% (2025) compared with other treatments. There were differences between the two years at the R1 and R3 stages. In 2024, there were no significant differences among the five groups, while in 2025, at the R1 stage, the T3 treatment was the highest among the five groups, increasing by 12.82%-26.02% compared with other treatments; at the R3 stage, the CK treatment was significantly lower than other treatments by 11.92%-14.50%. For GOGAT enzyme activity (**Figures 5e, f**), the T2 treatment increased by 6.90%-15.21% (2025) at the V12 stage and by 10.28%-24.06% (2025) at the R1 stage compared with other treatments. Except for periods without significant differences, the T1 treatment had lower enzyme activity than other treatments.

As shown in **Figure 5**, regarding glutamic oxaloacetic transaminase (GOT) activity (**Figures 5g, h**), the T2 treatment consistently exhibited the highest enzyme activity at the V12 stage, showing significant increases of 6.71%-21.67% (2024) and 8.95%-21.60% (2025) compared to other treatments. At the R1 stage, enzyme activity in T2, T3, and T4 treatments exceeded that of T1 and CK treatments. At the R3 stage, T3 treatment exhibited the highest enzyme activity, showing a significant increase of 3.92%-15.25% (2024) compared to other treatments. Regarding glutamic pyruvic transaminase (GPT) activity (**Figures 5i, j**), T1 treatment exhibited lower enzyme activity than other treatments during both V12 and R1 periods. In 2024, T2 treatment showed the highest activity, increasing by 4.02%-25.31% (V12) and 1.82%-21.41% (R1). In 2025, T3 treatment exhibited the highest activity, increasing by 6.00%-37.25% (V12). During the R3 period, T1 and T3 treatments showed the highest activity, increasing by 16.42%-38.03% and 9.23%-29.50% respectively (2025). Regarding glutamate dehydrogenase (GDH) activity (**Figures 5k, l**), T1 treatment consistently showed lower activity than other treatments across all three periods in 2024. In 2025, T1 treatment remained lower than others during the V12 period, while T2 treatment exceeded others, increasing by 2.12%-8.22%. During the R1 stage, T3 and T4 treatments exceeded others, increasing by 3.00%-8.76% and 3.11%-8.87%, respectively. No significant differences were observed among the five groups during the R3 stage. Among the above enzyme activities, the differences between the treatment groups were mainly observed at the V12 and R1 stages, with the T2 treatment of combined organic and chemical fertilizers being the best, and at the R3 stage, the T3 treatment also showed higher activity in some enzymes.

### 3.4 Leaf senescence process

**Figure 6:**
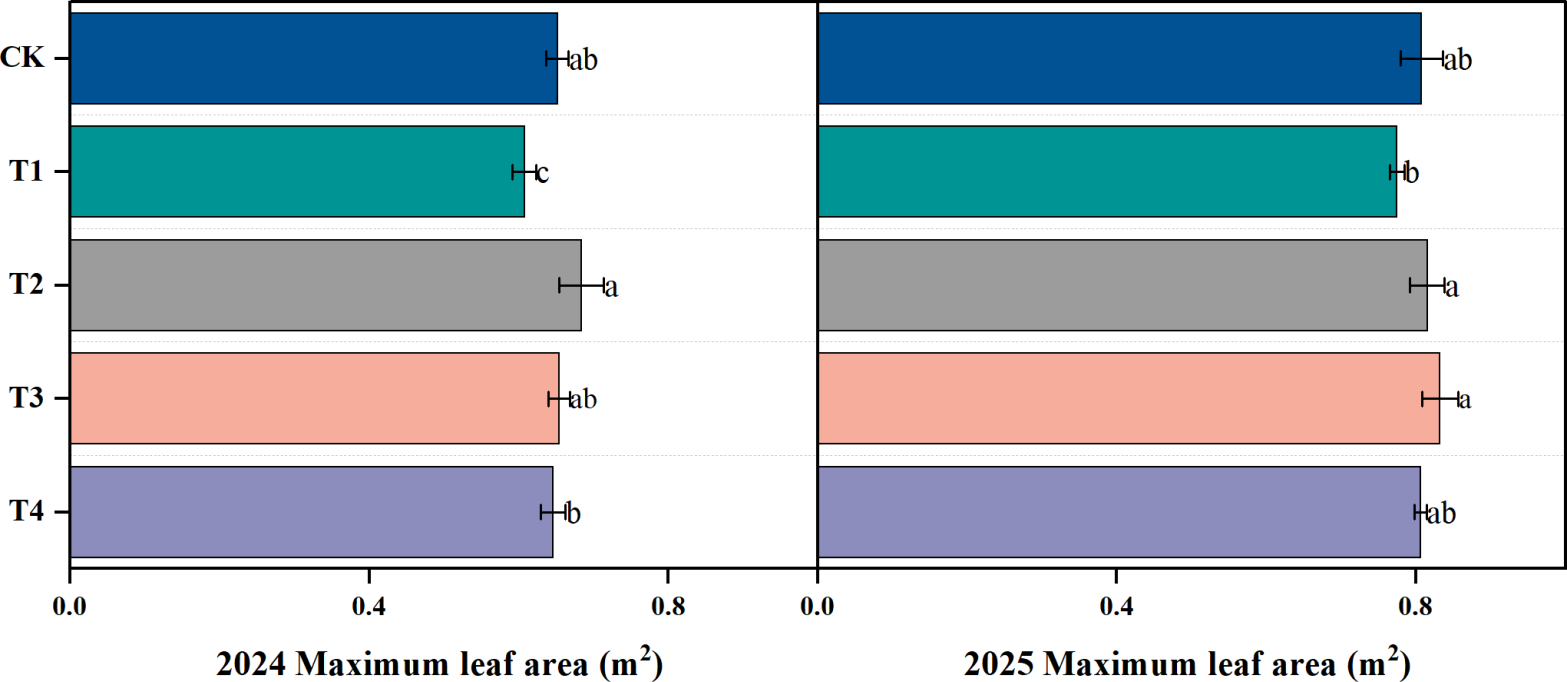
Differences in the management of R1 stage LAI. Different letters indicate significant differences between different treatments (P < 0.05).

As shown in **Figure 6**, significant differences existed in leaf area during the VT stage of maize among different treatments (*P* < 0.05). Over the two years, treatments T2 and T3 demonstrated advantages across all five groups. Specifically, treatment T2 significantly increased leaf area by 4.94%-12.56% (2024) and 0.93%-5.21% (2025) compared to CK, T1, and T4 treatments. T3 treatment significantly increased leaf area by 0.34%-7.62% (2024) and 3.05%-7.41% (2025) compared to CK, T1, and T4 treatments. Compared to CK treatment, T2 and T3 treatments with lower organic fertilizer substitution rates exhibited higher LAI, while treatments with complete organic fertilizer substitution showed decreased LAI.

**Figure 7:**
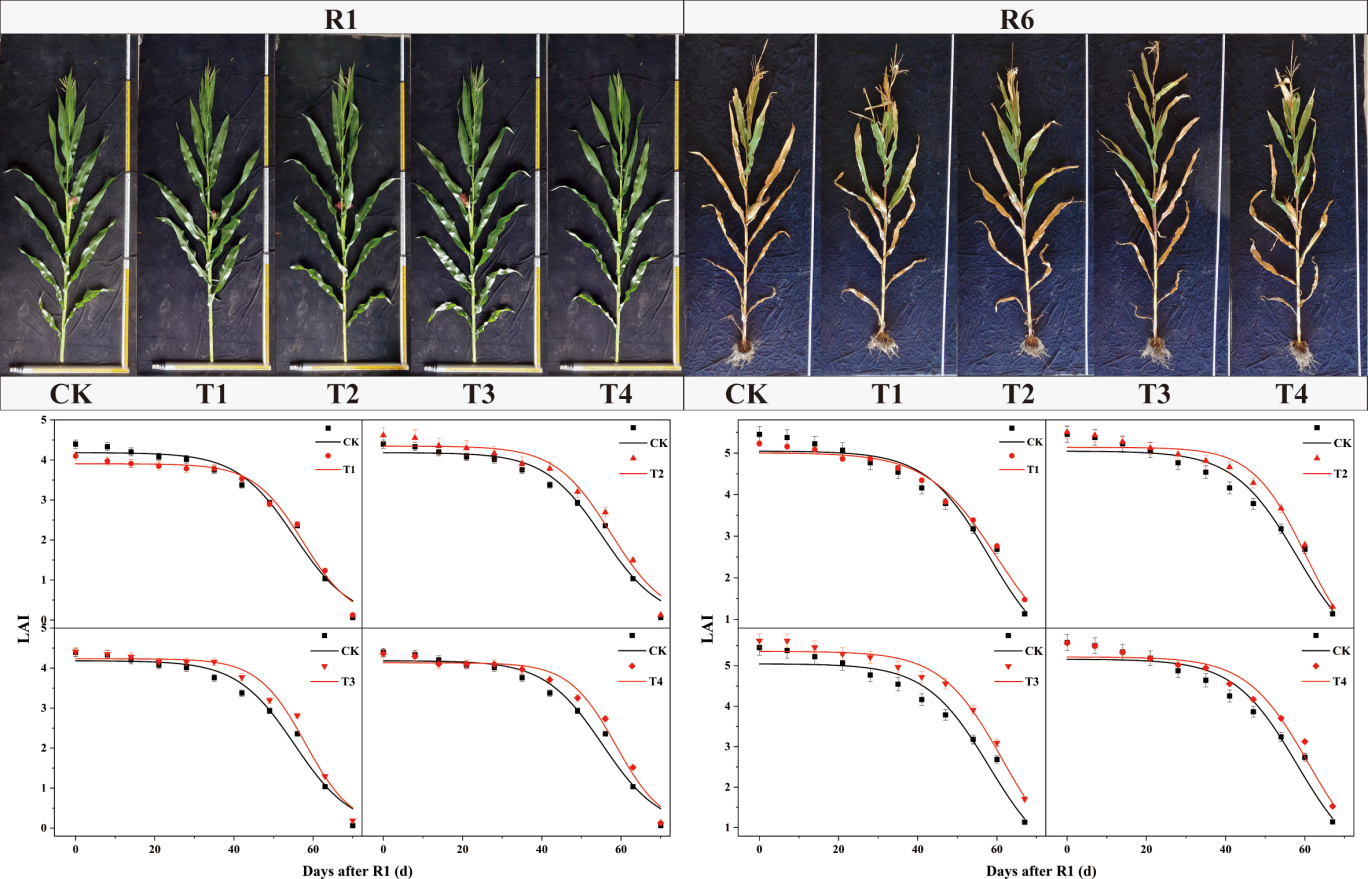
Differences in LAI over time following R1 across different treatments.

Monitoring the trend in green leaf area index (LAI) of plants after flowering (**Figure 7**) revealed that the fitted curves for LAI in maize post-flowering exhibited a parabolic pattern across all treatments. This manifested as a gradual decline initially followed by a steep decrease later on, though distinct trends emerged among the different treatments. Compared to the CK treatment, the fitted curves for T2, T3, and T4 treatments all delayed the initial rate of senescence and increased LAI during the late senescence stage. The T1 treatment also delayed the decline in LAI during late senescence. However, LAI in the T1 treatment was often lower than that in the CK treatment during the R1 stage, resulting in overall lower LAI during the early senescence stage of leaves compared to the CK treatment. This made it difficult to increase LAI during late senescence.

As shown in **Table 1**, the Logistic equations for each treatment were obtained through fitting, with correlation coefficients R² ranging from 0.9639 to 0.9802 (2024) and 0.9751 to 0.9926 (2025). Both years’ data fully reflected the leaf senescence processes under the following treatments. Significant differences were observed among treatments in both the time to reach maximum senescence rate (c) and the maximum senescence rate (V_max_). Regarding the time to reach maximum senescence rate (c), the CK treatment exhibited a lower c value than the other four treatments. The four treatments applying organic fertilizer all delayed the onset of maximum senescence rate. Among them, the c value of the CK treatment decreased by 3.68%-5.84% (2024) compared to the other treatments. The c value of the T3 treatment increased by 3.27%-5.64% (2025) compared to the other treatments; in terms of the maximum senescence rate (V_max_), the CK treatment had the lowest rate in 2024, decreasing by 7.47%-18.39% (2024) and 10.02%-25.69% (2025) compared to the other treatments, respectively. Organic fertilizer application primarily influenced maize leaf area by affecting LAI during the VT stage and delaying the onset of maximum senescence rate during leaf senescence. The treatment fully replacing chemical fertilizer with organic fertilizer reduced the maximum maize leaf area, while treatments with lower organic fertilizer replacement ratios (T2 and T3) in the organic-chemical fertilizer combination effectively increased maize leaf area.

**Table 1:** Logistic equations and their parameter descriptions for different treatments of post-flowering LAI dynamic changes.

Year	Treatments	Fitting curve equation	a	b	c	V_max_	R^2^
2024	CK	$\text{y}=4.18/(1+{\text{e}}^{\left(0.14-7.74\right)})$	4.18 ± 0.12	-0.14 ± 0.02	55.27 ± 1.19	-0.1442	0.9709
	T1	$\text{y}=3.91/(1+{\text{e}}^{\left(0.16\text{x}-9.19\right)})$	3.91 ± 0.08	-0.16 ± 0.02	57.45 ± 0.85	-0.1568	0.9802
	T2	$\text{y}=4.35/(1+{\text{e}}^{\left(0.14\text{x}-8.03\right)})$	4.35 ± 0.12	-0.14 ± 0.02	57.39 ± 1.22	-0.1559	0.9639
	T3	$\text{y}=4.23/(1+{\text{e}}^{\left(0.17\text{x}-9.87\right)})$	4.23 ± 0.09	-0.17 ± 0.02	58.04 ± 0.9	-0.1767	0.9759
	T4	$\text{y}=4.13/(1+{\text{e}}^{\left(0.17\text{x}-9.98\right)})$	4.13 ± 0.09	-0.17 ± 0.02	58.70 ± 0.95	-0.1743	0.9724
2025	CK	$\text{y}=5.05/(1+{\text{e}}^{\left(0.13\text{x}-7.33\right)})$	5.05 ± 0.21	-0.13 ± 0.02	57.96 ± 1.28	-0.1597	0.9751
	T1	$\text{y}=5.01/(1+{\text{e}}^{\left(0.11\text{x}-6.87\right)})$	5.01 ± 0.12	-0.11 ± 0.01	59.92 ± 0.78	-0.1437	0.9876
	T2	$\text{y}=5.14/(1+{\text{e}}^{\left(0.15\text{x}-9.04\right)})$	5.14 ± 0.11	-0.15 ± 0.01	60.07 ± 0.61	-0.1933	0.9926
	T3	$\text{y}=5.36/(1+{\text{e}}^{\left(0.13\text{x}-7.91\right)})$	5.36 ± 0.10	-0.13 ± 0.01	61.42 ± 0.56	-0.1725	0.9925
	T4	$\text{y}=5.10/(1+{\text{e}}^{\left(0.13\text{x}-7.87\right)})$	5.10 ± 0.15	-0.13 ± 0.02	60.84 ± 0.88	-0.1651	0.9806

x represents the number of days (d) since silk production, y denotes LAI, a is the theoretical initial value of LA, b is the constant describing the rate of senescence, c is the time required to reach the maximum senescence rate, and V_max_ is the maximum rate of senescence during the leaf senescence process, R² is the coefficient of determination.

### 3.5 Grain filling characteristics

**Figure 8:**
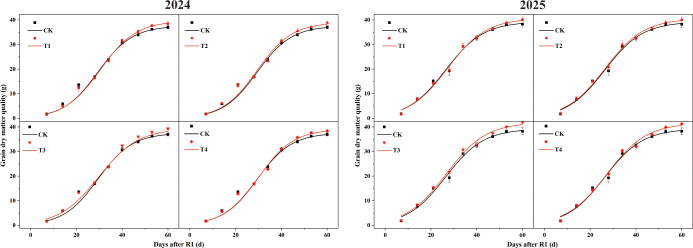
Logistic fitting curve for grain filling under different treatments.

As shown in **Figure 8**, the fitted curves for all five treatments over the two-year period exhibited an S-shaped pattern, characterized by a slow initial rate, rapid increase in the middle phase, and a subsequent return to a gradual rate. Differences were observed among the various treatments. Compared to the CK treatment, the four organic fertilizer application treatments all resulted in increased final grain dry matter accumulation. The increases ranged from 3.20%-5.76% (2024) and 4.73%–8.68% (2025) relative to the CK treatment. Among all treatments, T1, T2, T3, and T4 consistently exhibited significantly higher grain dry matter accumulation rates than the CK treatment around 40 d.

As shown in **Table 2**, the fitted Logistic equations for each treatment yielded correlation coefficients R² ranging from 0.9833 to 0.9957 (2024) and 0.9890 to 0.9924 (2025), which adequately reflected the grain dry matter accumulation process under the following treatments. Comparing the time required to reach maximum grain filling rate (T_max_) revealed that T1, T3, and T4 treatments exhibited higher T_max_ values than the CK treatment in both years, while the T_max_ value for treatment T2 was also higher than CK during 2025. Regarding the 100-kernel weight at maximum filling rate (W_max_), CK showed lower values than the four organic fertilizer treatments in both years. For the maximum filling rate (R_max_), all treatments except T3 in 2024 exceeded CK. The active filling period (P) was longer for all four organic fertilizer treatments than for CK. It is evident that although the CK treatment exhibited a higher grain filling rate, its active grain filling period was too brief, reaching maximum filling rate too rapidly, resulting in insufficient dry matter accumulation in the grains. In contrast, the T3 treatment, which achieved higher grain accumulation, maintained an active grain filling period longer than other treatments. This extended filling duration ultimately led to greater dry matter accumulation in the grains.

**Table 2:** Differences in grain filling parameters among different treatments.

Year	Treatments	a	b	k	T_max_	W_max_	R_max_	P	R^2^
2024	CK	37.56 ± 1.32	49.88 ± 4.61	0.13 ± 0.00	29.39	18.78	5.00	45.10	0.9957
	T1	39.58 ± 1.36	48.87 ± 6.46	0.13 ± 0.01	30.45	19.79	5.06	46.97	0.9952
	T2	38.91 ± 1.36	47.98 ± 6.81	0.13 ± 0.01	29.35	19.45	5.13	45.50	0.9943
	T3	39.34 ± 4.07	31.72 ± 8.2	0.12 ± 0.01	29.76	19.67	4.57	51.65	0.9833
	T4	39.11 ± 1.97	51.79 ± 8.03	0.13 ± 0.01	30.08	19.55	5.13	45.72	0.9971
2025	CK	39.25 ± 1.23	23.88 ± 6.84	0.12 ± 0.01	26.37	19.63	4.72	49.86	0.9890
	T1	40.64 ± 1.17	24.77 ± 6.21	0.12 ± 0.01	27.22	20.32	4.79	50.88	0.9917
	T2	40.51 ± 1.08	22.53 ± 5.2	0.12 ± 0.01	26.52	20.26	4.76	51.09	0.9924
	T3	41.86 ± 1.31	21.5 ± 5.56	0.11 ± 0.01	26.69	20.93	4.81	52.19	0.9901
	T4	41.59 ± 1.34	22.94 ± 6.07	0.12 ± 0.01	27.22	20.79	4.79	52.14	0.9901

a represents the final grain dry matter growth amount, b is the parameter of the fitting equation, k signifies the growth rate parameter, T_max_ indicates the time required to reach the maximum grain filling rate (d), W_max_ denotes the 100-kernel weight at the maximum grain filling rate (g), R_max_ represents the maximum grain filling rate (g/d), and P indicates the active grain filling period (d), R² is the coefficient of determination.

### 3.6 Accumulation, allocation, and transport of dry matter

#### 3.6.1 Dry matter accumulation

As shown in **Table 3**, there were significant differences in dry matter accumulation among treatments at different stages (*P* < 0.05). Over the two years, except for the V6 stage in 2024, the trend at the V6 and V12 stages was T2 > CK, T3, T4 > T1. Specifically, at the V6 stage in 2024, the T2 and CK treatments increased by 9.62%-38.83% and 9.78%-39.04%, respectively, compared with other treatments; at the V12 stage, the T2 treatment increased by 10.30%-42.21% compared with other treatments. In 2025, the T2 treatment increased by 10.84%-73.11% (V6) and 9.05%-56.18% (V12). At the R1, R3, and R6 stages in 2025, the T3 treatment showed significant differences compared with other treatments, increasing by 4.46%-13.49% (R1), 4.03%-15.51% (R3), and 4.91%-12.82% (R6) relative to the other four treatments. At the R3 stage in 2024, the T3 treatment increased by 5.84%, 15.04%, and 2.69% compared with CK, T1, and T4, while the T2 treatment increased by 6.45%, 15.70%, and 3.48% compared with CK, T1, and T4. In other stages, the T1 treatment was often significantly lower than other treatments. Thus, during the vegetative growth stages V6 and V12, the treatment with organic fertilizer replacing 10% of chemical fertilizer (T2) showed the best growth effect, while during the reproductive growth stages after R1, the treatment with organic fertilizer replacing 20% of chemical fertilizer (T3) was most beneficial for dry matter accumulation.

**Table 3:** Differences in dry matter accumulation under different treatments at different stages.(kg/ha).

Year	Treatments	V6	V12	R1	R3	R6
2024	CK	603.45 ± 59.05a	5319.45 ± 231.28b	11532.60 ± 756.03a	17724.04 ± 511.38b	22196.93 ± 1297.12a
	T1	434.03 ± 28.07c	4125.83 ± 124.95c	10207.58 ± 750.3b	16306.54 ± 434.58c	19388.25 ± 882.85b
	T2	602.55 ± 15.99a	5867.33 ± 404.62a	11925.00 ± 602.13a	18866.48 ± 163.43a	23899.05 ± 586.77a
	T3	549.68 ± 25.65ab	5311.13 ± 48.23b	11473.88 ± 417.02a	18759.49 ± 615.77a	23281.20 ± 1186.72a
	T4	529.88 ± 17.59b	5096.7 ± 109.7b	11483.33 ± 391.26a	18267.75 ± 281.56ab	22574.25 ± 769.81a
2025	CK	645.53 ± 94.17ab	4598.55 ± 34.69ab	12048.08 ± 542.79ab	24309.56 ± 300.26bc	27454.50 ± 736.05ab
	T1	413.33 ± 20.26c	4269.15 ± 69.84b	11350.58 ± 716.80b	23997.94 ± 1522.47c	26691.75 ± 1665.97b
	T2	715.50 ± 45.29a	4745.25 ± 243.00a	12332.48 ± 681.38ab	25866.79 ± 751.39ab	28642.61 ± 1719.57ab
	T3	591.98 ± 27.43b	4653.68 ± 284.73ab	12882.26 ± 971.35a	27718.99 ± 1253.55a	30114.34 ± 1360.35a
	T4	591.75 ± 9.84b	4589.10 ± 246.43ab	12190.61 ± 610.50ab	25914.15 ± 473.19ab	28705.61 ± 1380.03ab

Different letters indicate significant differences between different treatments (*P* < 0.05). The same applies below.

#### 3.6.2 Dry matter distribution

**Figure 9:**
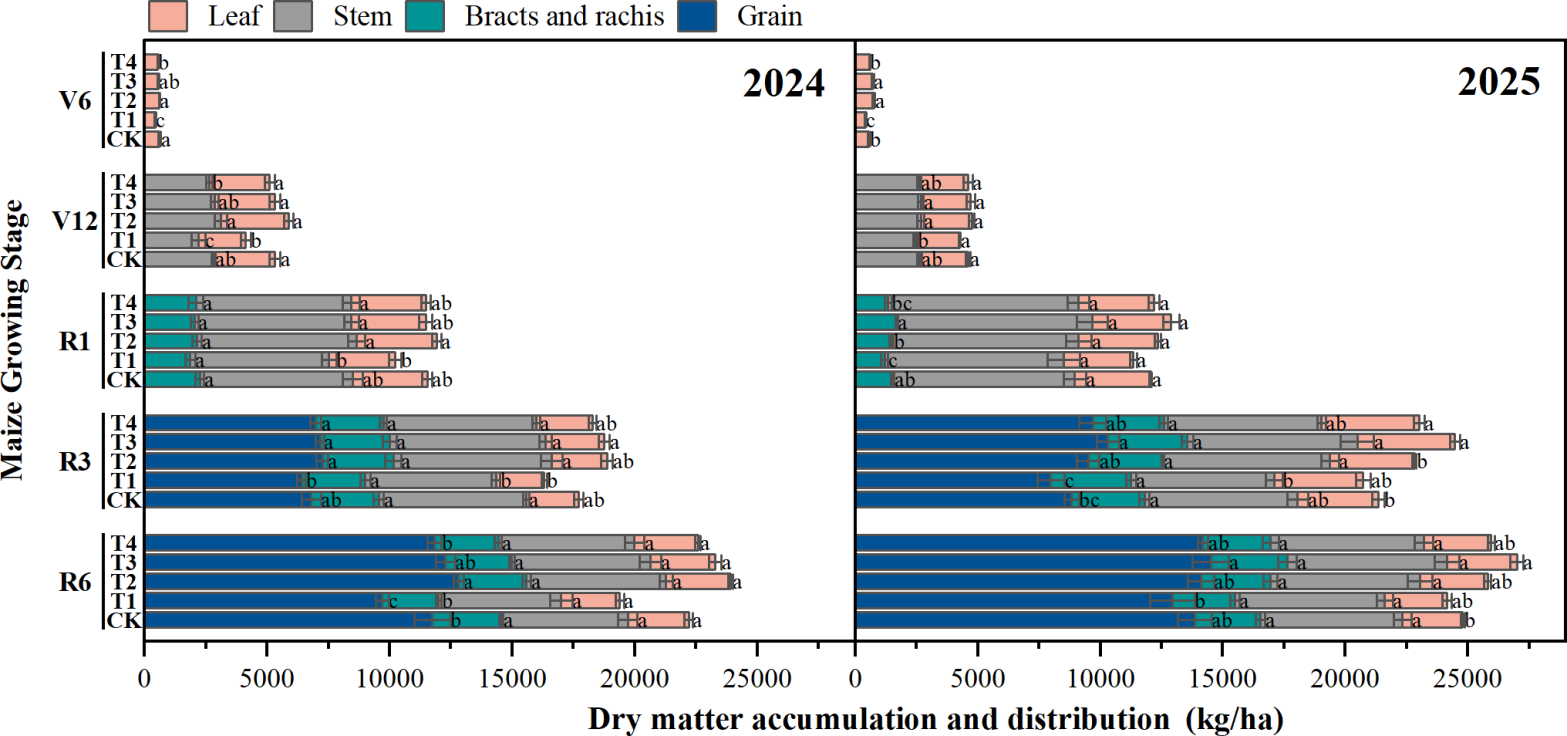
Differences in dry matter allocation among treatments at different growth stages. Different letters indicate significant differences between different treatments (P < 0.05).

As shown in **Figure 9**, the five treatments exhibited significant differences (*P* < 0.05) in dry matter accumulation across various maize organs at different growth stages. In terms of leaf dry weight, during the V6 stage, the T2 treatment showed higher values in both years, increasing by 9.62%-38.83% (2024) and 27.76%-82.91% (2025) compared to the other treatments. Significant differences existed between treatments at the V12 and R1 stages in 2024 but not in 2025. At the V12 stage, T1 treatment reduced leaf dry weight by 21.20%-29.84% compared to other treatments. At the R1 stage, T2 treatment increased leaf dry weight by 6.83%-21.79% compared to other treatments. At stages R3 and R6, T3 treatment consistently showed higher accumulation. At R3, T3 treatment increased by 5.46%-22.01% (2024) and 9.47%-20.63% (2025) compared to other treatments. At R6, T3 treatment increased by 3.49%-17.06% (2025). Regarding stem weight, patterns at the V6 and V12 stages mirrored those observed for leaf dry weight. At the V6 stage, the T3 treatment in 2024 showed increases of 9.28%-41.92% compared to other treatments, while in 2025, it increased by 2.33%, 9.09%, and 2.85% relative to the CK, T1, and T4 treatments, respectively. At the V12 stage, the T1 treatment showed a decrease of 9.26%-13.01% compared to other treatments in 2024. At the R3 stage in 2024, the T1 treatment was lower than other treatments, decreasing by 11.52%-17.40%. In 2025, the T2 and T3 treatments were higher than other treatments, increasing by 6.05%-16.82% and 7.93%-18.89%, respectively. No significant differences were observed in the R6 stage. For glumes and rachis, no significant differences were found except for T3 treatment being significantly higher than others in the R1 stage in 2025. For grain dry weight, T1 treatment was lower than others in both R3 and R6 stages. In 2025, T3 treatment showed higher grain dry weight than others, increasing by 6.31%-29.07% (R3) and 2.34%-12.15% (R6), while in 2024, the T2 treatment was higher than other treatments, increasing by 4.39%-32.15% (2024).

The trend of leaf and stem dry matter accumulation in the V6 and V12 stages of each treatment group is similar to that of the whole plant. Treatment T2, which uses organic fertilizer instead of chemical fertilizer with a smaller proportion, has a better effect. However, after entering the R1 stage, the dry matter in the reproductive organs and grain parts is more affected by the year, and the accumulation of T2 and T3 treatments is greater.

#### 3.6.3 Dry matter transport

As shown in **Table 4**, there were significant differences (*P* < 0.05) in the contribution of pre-flowering leaves and stems to dry matter yield among the treatment groups. Among the five treatments, CK and T2 treatments had higher leaf translocation amount and leaf translocation rate than other treatments, with CK treatment being the most significant, increasing leaf translocation amount by 17.70%-87.51% (2024) and 43.75%-98.35% (2025) and leaf translocation rate by 3.03%-7.45% (2024) and 6.95%-10.30% (2025). For stem-sheath translocation amount and stem-sheath translocation rate, CK treatment was significantly lower than other treatments. A similar trend was observed in the post-anthesis dry matter accumulation contribution rate. The four treatments with organic fertilizer application all increased post-anthesis dry matter accumulation, and CK treatment decreased by 2.60%-4.64% (2024) 2.24%-4.80% (2025). Among the five treatments, CK treatment had the highest leaf and stem-sheath translocation amounts and the lowest post-anthesis dry matter accumulation contribution rate, while T1 showed the opposite, overall exhibiting a higher post-anthesis dry matter contribution. The three treatments with combined application of organic and chemical fertilizers fell between the two, and the application of organic fertilizer expanded the proportion of post-anthesis dry matter accumulation.

**Table 4:** Differences in dry matter transport between different treatments.

Year	Treatments	DMTA (kg/ha)		DMTE (%)		CRDMG (%)
		Leaf	Stem	Leaf	Stem	
2024	CK	564.08±32.28ab	1076.85±9.19a	18.52±0.46a	17.31±1.02a	86.00±0.59b
	T1	300.83±86.56c	652.50±186.25b	11.07±2.36b	11.63±3.84b	90.15±1.40a
	T2	654.75±119.42a	807.75±160.63b	19.93±2.69a	12.38±2.08b	88.60±2.01a
	T3	391.28±60.11bc	753.53±159.41b	12.91±1.95b	11.85±3.04b	90.65±1.34a
	T4	479.25±161.3abc	768.83±61.77b	15.50±4.53ab	12.20±1.64b	89.44±1.32a
2025	CK	652.05±72.03a	1617.30±145.68a	21.08±2.24a	21.77±1.36a	83.60±0.36c
	T1	328.73±70.24b	1178.33±331.55b	11.58±2.94b	15.97±3.00b	88.41±1.26a
	T2	453.60±74.26b	1540.24±19.58ab	14.13±2.45b	20.20±1.15ab	85.84±0.81b
	T3	347.85±71.65b	1474.76±152.29ab	10.78±1.26b	18.45±1.02ab	87.47±0.98ab
	T4	356.06±99.93b	1484.78±170.62ab	11.40±2.37b	19.15±1.88ab	87.03±1.12ab

Note: DMTA is the dry matter translocation amount, DMTE is the dry matter translocation efficiency, CRDMG is the contribution rate of dry matter to grains.

### 3.7 Correlation analysis of production volume and its constituent factors

#### 3.7.1 Maize yield and its components

As shown in **Table 5**, significant differences (*P* < 0.05) were observed among treatments for both 100-kernel weight and kernels per ear, while no significant differences were found in the number of ears. In terms of 100-kernel weight, the CK treatment consistently yielded lower values than other treatment groups over both years, reducing 100-kernel weight by 3.20%-5.76% (2024) and 4.71%-8.68% (2025). Regarding ears number, the T1 treatment was significantly lower than other treatments, reducing kernels per ear by 6.40%-11.91% (2024) and 3.18%-5.81% (2025). Inter-annual variations were observed in both 100-kernel weight and kernels per ear. In 2024, no significant differences existed among treatments except for CK. In 2025, T3 and T4 treatments significantly exceeded others, increasing kernel weight by 4.32%-9.50% and 2.87%-7.98% compared to CK, T1, and T2, respectively. In 2024, T2 treatment yielded the highest kernels per ear, increasing by 2.96%-13.52% compared to other treatments. In 2025, the trend showed T3, T2, T4 > CK > T1, with T3 treatment being the highest, increasing by 1.13%-6.17% compared to other treatments.

**Table 5:** Differences in yield composition factors among different processing methods.

Year	Treatments	Ear number (ear/ha)	100-kernel weight (g)	Kernels per ear
2024	CK	65934 ± 2537.80a	37.02 ± 0.67b	489 ± 8.08ab
	T1	62088 ± 2104.24a	38.48 ± 0.76a	449 ± 15.01c
	T2	63736 ± 4395.60a	38.87 ± 0.47a	509 ± 16.65a
	T3	64469 ± 4575.09a	39.29 ± 0.57a	495 ± 8.08ab
	T4	65934 ± 2197.80a	38.25 ± 0.58a	479 ± 17.01b
2025	CK	66645 ± 4362.41a	38.26 ± 1.37b	609 ± 9.71ab
	T1	65325 ± 4680.80a	40.16 ± 1.18ab	589 ± 21.94b
	T2	65700 ± 323.32a	40.15 ± 1.02ab	619 ± 5.03a
	T3	67235 ± 2338.48a	41.89 ± 2.08a	626 ± 17.50a
	T4	67960 ± 2942.29a	41.31 ± 1.82a	618 ± 11.27a

Different letters indicate significant differences between different treatments (P < 0.05).

**Figure 10:**
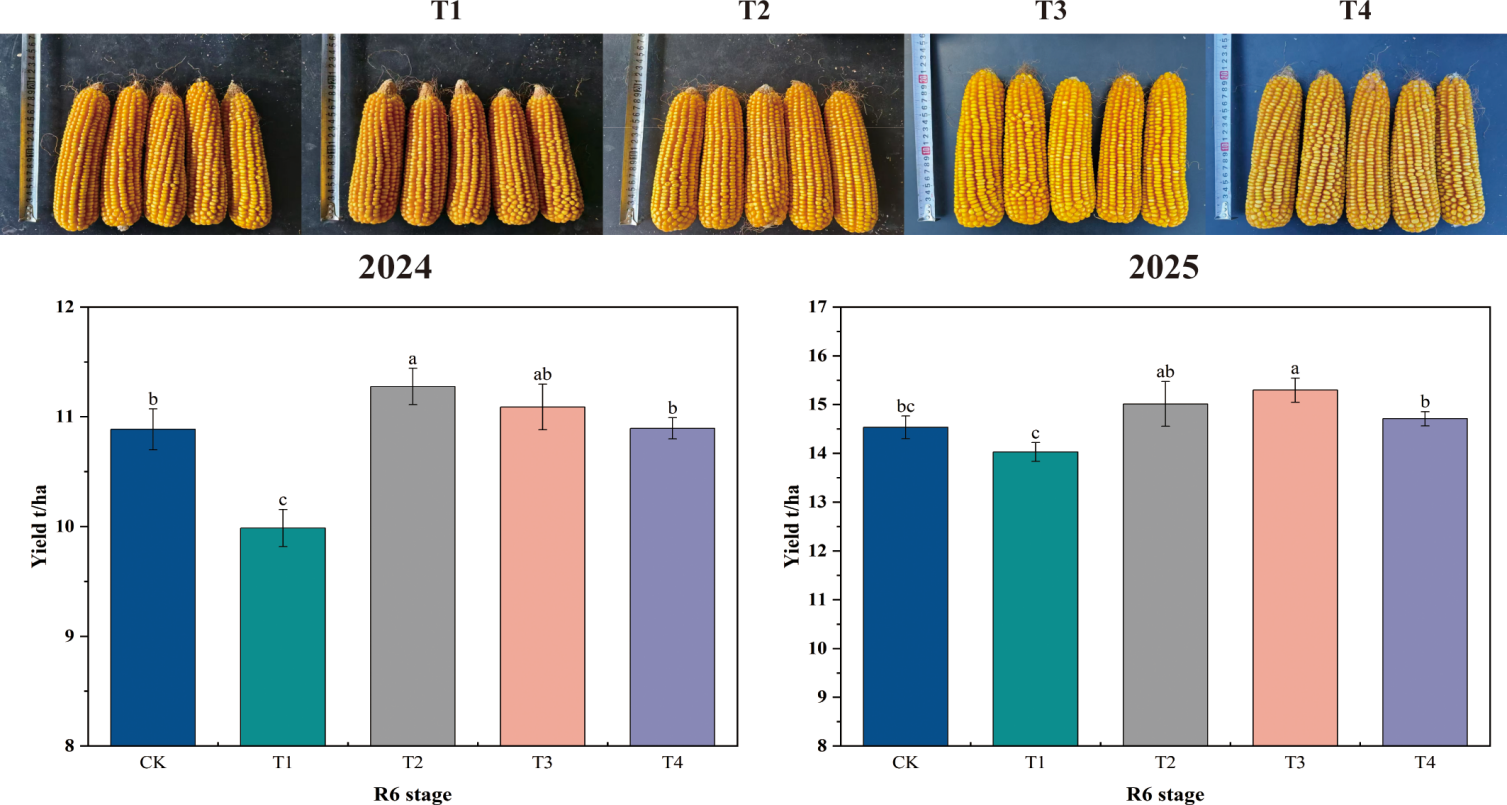
Differences in maize yield among treatment groups. Different letters indicate significant differences between different treatments (P < 0.05).

In terms of yield (**Figure 10**), the trend over the two years showed T2 and T3 > T4, and CK > T1 treatments. In 2024, the T2 treatment was significantly higher than the other treatments, increasing by 3.58%, 12.91%, 1.68%, and 3.50% compared to CK, T1, T3, and T4 treatments, respectively. While in 2025, the T3 treatment was significantly higher than the others, increasing by 5.25%, 9.04%, 1.89%, and 3.98% compared to the CK, T1, T3, and T4 treatments, respectively. Different treatments had varying effects on 100-kernel weight, kernels per ear, and yield. The CK treatment reduced 100-kernel weight, while the T1 treatment decreased the kernels per ear. However, the three treatments combining organic and chemical fertilizers (T2, T3, T4) maintained higher levels of 100-kernel weight and kernels per ear, ultimately boosting yield. Among these, the T2 and T3 treatments, which replaced 10% and 20% of chemical fertilizer with organic fertilizer respectively, demonstrated the most favorable effects.

#### 3.7.2 Correlation analysis of yield-related factors and yield

**Figure 11:**
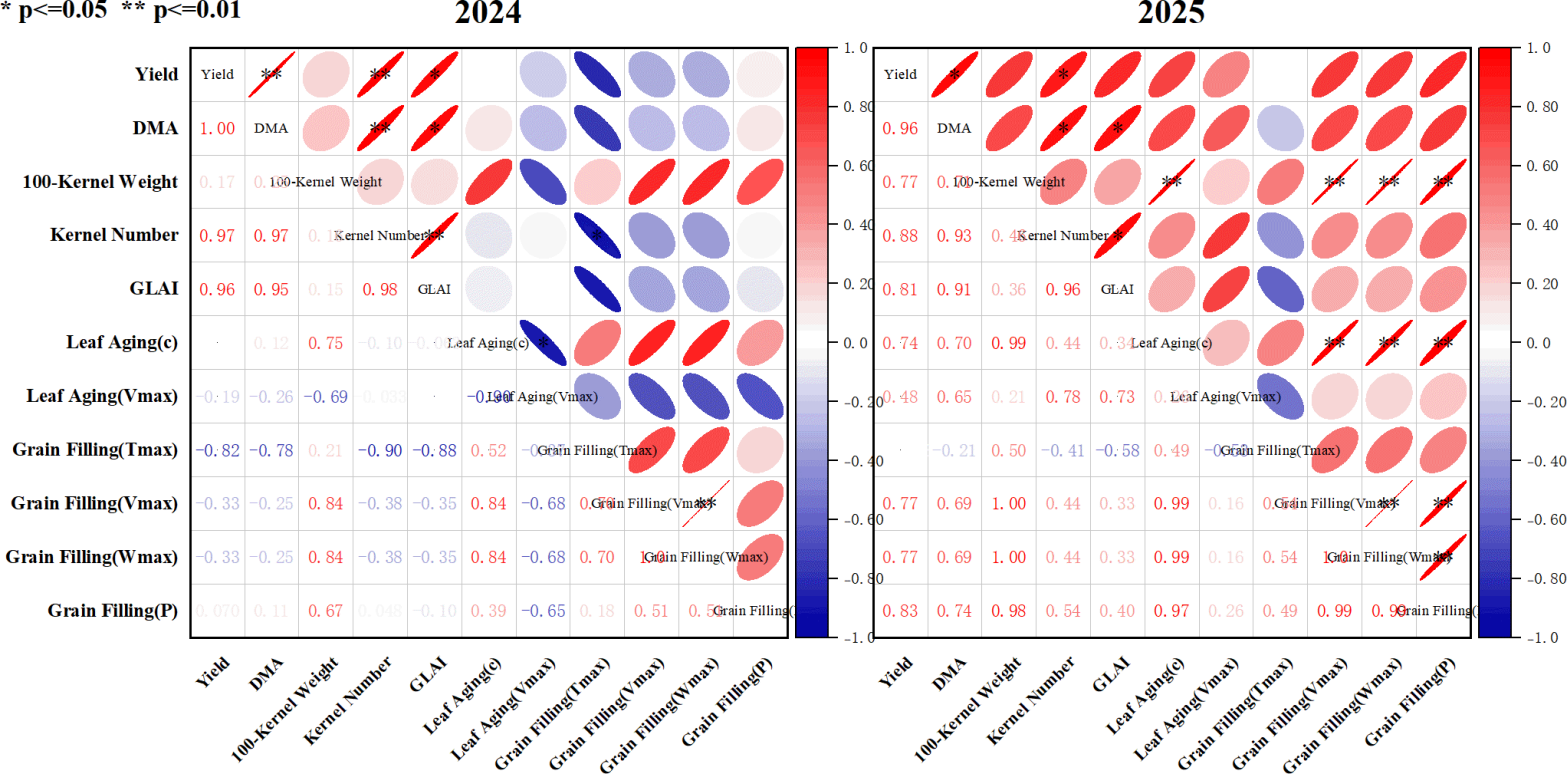
Correlation between mature maize yield and related factors.

As illustrated in **Figure 11**, analysis of yield-related factors across two years revealed that dry matter accumulation and grain number per ear at maturity exhibited the most direct correlation with yield. This correlation was strongly significant in 2024 (*P* < 0.01) and significantly significant in 2025 (*P* < 0.05). Secondarily, the LAI during the R1 stage also exhibited a positive correlation with yield, showing significant correlation in 2024(*P* < 0.05). LAI significantly influenced both the kernels per ear and dry matter accumulation at maturity. In 2025, positive correlations (*P* < 0.05) were also observed between 100-kernel weight and the number of days to maximum leaf senescence rate (c), maximum grain filling rate (V_max_), 100-kernel weight at maximum grain filling rate (W_max_), and active grain filling period (P).

## 4 Discussion

### 4.1 Regulating photosynthetic pigments in leaves to enhance light energy utilization

Chlorophyll is a crucial photosynthetic pigment that absorbs light energy during plant photosynthesis, participating in light energy collection and transfer. Its concentration directly indicates the strength of plant photosynthetic performance. The SPAD value determines relative chlorophyll content by measuring light intensity at two wavelengths: 650 nm and 940 nm (Szulc et al., 2021). Analysis revealed that SPAD values exhibited distinct advantages at different growth stages: during the early stages (V6, V12, R1, R3), the treatment with the lowest organic fertilizer substitution rate (T2) showed significant advantages, while in the later stages, the treatment with a moderate organic fertilizer substitution rate (T3) demonstrated significant advantages. In contrast, the treatment applying only chemical fertilizers (T1) showed decreased SPAD values. Previous research suggests that chemical fertilizers provide crops with higher initial nutrient levels, while organic fertilizers exhibit greater nutrient availability during the middle and late growth stages (Xu, 2001). These findings are consistent with the results of this study.

Chlorophyll fluorescence is a direct reflection of energy conversion and dissipation in plant photosynthesis (Butler and Kitajima, 1975). Under environmental stress, the distribution of absorbed light energy shifts, thereby altering fluorescence dynamics. Among chlorophyll fluorescence parameters, PSII maximum photochemical efficiency (*F_v_/F_m_*) and actual photochemical efficiency *Y(II)* reflect the efficiency of photosystem II photochemistry and are commonly used to assess plant physiological status (Ansari et al., 2022). This study found no significant differences in *F_v_/F_m_* among all treatment groups. However, significant differences in *Y(II)* were observed only during the vegetative growth stages V6 and V12. Overall, three gradients emerged: the two treatments with high chemical fertilizer application (CK, T2), the two treatments combining organic and chemical fertilizers (T3, T4), and the treatment using only organic fertilizer (T1). Many studies believe that the application of organic fertilizer has a slow-release effect, and because the nitrogen, phosphorus and other elements in organic fertilizer either mainly exist in organic form or are tightly combined with other minerals (Rajput et al., 2022; Rossi et al., 2023). The short-term impact of organic fertilizer on soil nutrients, especially in the topsoil, may not be significant (Samson et al., 2021), and the insufficient nutrients may have an inhibitory effect on maize leaves during the rapid growth stage. Nutrient deficiencies could thus inhibit maize leaf growth during rapid development stages. Notably, the *F_v_/F_m_* ratio remains relatively stable under mild stress but declines significantly under severe stress (Jiao and Hu, 2025), consistent with the findings of this study. The non-photochemical quenching coefficient (*NPQ*) reflects the proportion of light energy absorbed by PSII antenna pigments that cannot be utilized for photosynthetic electron transport and is instead dissipated as heat. This study found differences among treatments during the V6 and R3 stages. During the V6 stage, the trend was opposite to that of *Y(II)*: reduced *Y(II)* led to increased unusable light energy, causing *NPQ* to rise and resulting in a significant increase in *NPQ* for the T1 treatment at the V6 stage. At the R3 stage, with no significant differences in *Y(II)*, the increase in *NPQ* was likely due to *NPQ* promoting the dissipation of excess absorbed light energy as heat, thereby protecting photosynthetic structures from oxidative damage (Murchie and Ruban, 2020).

### 4.2 Regulate leaf photosynthetic physiology and synergistic carbon nitrogen metabolism capacity

The absorption and utilization of CO_2_ depend on nitrogen metabolism within leaves. Nitrogen metabolism involves a series of physiological reactions, including nitrogen uptake, transport, reduction, and assimilation of amino acids (Li et al., 2024). It influences crop growth and development by participating in the synthesis of enzymes and photosynthetic pigments associated with carbon metabolism (Hu et al., 2021). Most plant species can utilize NH_4_^+^, NO_3_^-^, and urea as nitrogen sources. Nitrates are converted to ammonium salts via NR and nitrite reductase (NiR). Ammonium salts are assimilated into glutamine via GS, the first amino acid formed in nitrogen assimilation, and subsequently converted through GOGAT (Tschoep et al., 2009). These converted substances are utilized in various biosynthetic reactions (Yang et al., 2020). Insufficient inorganic nitrogen supply typically directly impacts the activity of enzymes involved in maize nitrogen metabolism. Studies indicate that combined organic-inorganic fertilizer application significantly increases the activity of enzymes related to leaf nitrogen metabolism and starch synthesis, such as nitrate reductase, (GS), sucrose synthase (SUS), and starch synthase (SS), thereby enhancing crop nutrient accumulation (Shi et al., 2017). This aligns with the findings of this study, which revealed that enzyme activity was generally higher in the treatment where organic fertilizer replaced 10% of chemical fertilizer (T2) at the V12 stage, while the treatment where organic fertilizer replaced 20% of chemical fertilizer (T3) performed better at the R3 stage. Enzyme activity in the treatment where organic fertilizer replaced 100% of chemical fertilizer (T1) was consistently lower than in other treatments. The key enzymes related to amino acid synthesis and decomposition in nitrogen metabolism, namely glutamic-oxaloacetic transaminase (GOT), glutamic-pyruvic transaminase (GPT), and glutamate dehydrogenase (GDH), also showed the same trend. The combined application of organic and chemical fertilizers enhances soil nutrient availability and plant uptake (Raza et al., 2022). Research indicates that replacing chemical fertilizers with organic fertilizers can directly reduce NH_3_ volatilization in farmland by lowering ammonium nitrate levels in the soil near roots (Xue, 2025). This enhances nitrogen use efficiency and increases leaf enzyme activity, thereby promoting carbon and nitrogen metabolism.

In C4 plant photosynthesis, the carbon assimilation mechanism is achieved by spatially separating the initial fixation of atmospheric CO_2_ from the Calvin cycle. CO_2_ is primarily fixed onto PEP, catalyzed by PEPC in mesophyll cells. C4 acids are transported to vascular sheath cells, where they supply CO_2_ to RuBP carboxylase (Sage, 2004; Doubnerová and Ryšlavá, 2011). Through photosynthetic CO_2_ fixation, RuBP carboxylase converts CO_2_ into sugars and lipids (Ashida and Yokota, 2011). Studies indicate that trends in photosynthetic enzyme activity during the vegetative growth stages (V12, R1, R3) align with fertilizer application rates across all groups. However, reproductive growth stages show more pronounced effects from the organic-fertilizer mixture ratios. Specifically, at the V12 stage, RuBP carboxylase activity was significantly differentiated among the five treatments: the control (CK) receiving only chemical fertilizer, the treatments where organic fertilizer replaced 10% and 20% of chemical fertilizer (T2, T3) were higher than the treatment where organic fertilizer replaced 30% of chemical fertilizer (T4), which was higher than the treatment using only organic fertilizer (T1). In contrast, for PEPC activity, the treatment using only organic fertilizer (T1) was significantly lower than the other treatments. The trends in differences between treatments were similar, but RuBP carboxylase activity exhibited greater sensitivity to treatment variations. Previous studies have shown that under different growth conditions, the relative increase in PEPC content in maize leaves with increasing nitrogen was significantly higher than that of RuBP carboxylase (Sugiyama et al., 1984). This aligns with the results of this study, where differences between treatments were more pronounced due to the low nitrogen allocation. Nitrogen exhibits higher transport efficiency in young maize leaves (Kumar et al., 2025), leading to more pronounced differences during the vegetative growth stage. As leaves begin to senesce at the R3 stage, the control treatment (CK) receiving only chemical fertilizer showed significantly lower RuBP carboxylase activity compared to treatments incorporating organic fertilizer. Research indicates that cultivating nitrogen-efficient varieties after organic fertilizer application significantly enhances GS, GPT, and GOT activities in flag leaves, while slowing the decline in antioxidant enzyme activity during the late growth stage (Zhang, 2016). This aligns with the findings of this study.

### 4.3 Enhance photosynthetic capacity and increase dry matter accumulation

Photosynthesis is a biological process through which plants (including photosynthetic bacteria) convert light energy into chemical energy to synthesize organic compounds. Enhancing photosynthesis is one of the most effective methods to improve crop quality and yield (Shi et al., 2022; Yang et al., 2022). This study found that the photosynthetic parameters at the V6 stage of maize had the most significant effects. During the V6 stage, maize undergoes rapid vegetative development, requiring photosynthesis to fix carbon dioxide and absorb nitrate from the soil to obtain the carbon and nitrogen sources needed to support stem elongation, leaf expansion, and root development (Yan et al., 2021). Differences in fertilizer application rates led to variations in nutrient uptake among treatments during the early growth stages. While chemical fertilizers provide higher initial nutrient levels, studies indicate that organic fertilizers exhibit greater nutrient availability during the middle and late growth stages (Xu, 2001). The application of chemical fertilizers compensates for the nutritional requirements of early crop growth. Analysis of photosynthetic parameters during the V12, R1, and R3 stages revealed that differences between treatments narrowed as the growth period progressed, consistent with previous studies.

The accumulation of dry matter also exhibited this trend: during the V6 and V12 stages, the highest accumulation occurred in the treatment where organic fertilizer replaced 10% of chemical fertilizer (T2), followed by the treatment using entirely chemical fertilizer (CK). Conversely, during the R1, R3, and R6 stages, the highest accumulation was observed in treatments where organic fertilizer replaced 10% and 20% of chemical fertilizer (T2 and T3). During fertilizer application, the availability of nutrients in the soil must align with crop demand to determine dry matter accumulation at each growth stage. If applied nutrients fall below requirements, plants cannot achieve maximum growth performance (Reyes et al., 2017). The treatment where organic fertilizer completely replaced chemical fertilizer (T1) significantly reduced maize dry matter accumulation across all growth stages, particularly during the vegetative growth phase before silking. Some studies have shown that under Mediterranean climatic conditions, the growth indices of sweet corn, such as leaf area index and dry matter accumulation, under the sole application of organic fertilizer are inferior to those under the sole application of chemical fertilizer, but superior under the combined application of organic and inorganic fertilizers (Efthimiadou et al., 2010). Conversely, on China’s North China Plain, applying a mixture of 15%-30% organic fertilizer and 70%-85% chemical fertilizer enhances nitrogen uptake and dry matter accumulation after grain filling (Zhai et al., 2024), consistent with the findings of this study.

### 4.4 Extend the post-flowering nutrient accumulation process and balance nutrient transport efficiency

Leaves, as the primary organs supplying carbohydrates essential for plant growth, exhibit aging rates that significantly influence final crop yields (Lukeba et al., 2013; Szabó et al., 2022). The application of partial organic fertilizers effectively enhances maize photosynthetic characteristics, prolonging the duration of photosynthetic activity in the crop population during the later growth stages compared to chemical fertilizer alone. This results in higher and more stable yields (He et al., 2019; Xu et al., 2021). This study found that all four treatments involving the application of organic fertilizers effectively delayed the aging process of maize leaves and postponed the time at which the maximum rate of leaf senescence occurred. Previous research indicates that deep vertical tillage combined with appropriate organic fertilizer significantly increases maize leaf area index (LAI) and SPAD values while delaying leaf senescence onset by 2.9-13.9 days (Li et al., 2026), consistent with the present findings. Excessive chemical nitrogen fertilizer application often causes a rapid decline in leaf area index during the late growth stage, hindering maize dry matter accumulation (Wu et al., 2011). However, while fully replacing chemical fertilizers with organic fertilizers can delay leaf senescence, it reduces the green leaf area index (LAI) during the silking stage due to insufficient nutrient accumulation in the early growth period, resulting in lower dry matter accumulation than the CK treatment. In contrast, the combined application of organic and chemical fertilizers significantly increases LAI during the silking stage and enhances post-flowering dry matter accumulation while delaying leaf senescence. With the treatment replacing 20% of chemical fertilizer with organic fertilizer (T3) yielding the best results.

Regarding grain filling characteristics, organic fertilizer not only prolongs the time required to reach maximum filling rate and extends the active filling period but also increases the 100-kernel weight at peak filling rate, effectively boosting kernel weight. Extending the period of leaf greenness and photosynthetic activity increases net photosynthetic rate per unit area, maintaining higher vitality during the late growth stage. This enhances the supply of photosynthetic products to grains, thereby improving grain filling rate (Liu et al., 2022). Analyses indicate that organic fertilizers significantly increased leaf area index, SPAD values, and dry matter weight during the grain filling and maturity stages overall, favoring grain filling (Liu et al., 2021a). Consistent with our findings, all four organic fertilizer treatments yielded higher final grain dry matter accumulation than the CK treatment. Analysis of grain filling rate and duration revealed that organic fertilizer application extended the active grain filling period and prolonged the time required to reach maximum filling rate, thereby increasing grain dry matter accumulation. Higher chlorophyll content, photosynthetic rate, and leaf area duration during the grain filling period are key factors in enhancing grain filling rate (Zhai et al., 2021).

Dry matter accumulation forms the foundation of maize yield formation, particularly the direct accumulation of photosynthetic products after tasseling (Liu et al., 2020). Research indicates that dry matter accumulation and transport in maize are dynamically regulated by nutrient supply (Zhang et al., 2020). Analysis of dry matter allocation and transport revealed that the control treatment (CK) with full chemical fertilizer application exhibited the lowest post-flowering dry matter contribution rate, while the treatment (T1) with full organic fertilizer substitution achieved the highest rate. Among the three treatments combining organic and chemical fertilizers, the 10% replacement rate treatment (T2) exhibited the lowest post-flowering dry matter contribution rate, while the 20% (T3) and 30% (T4) replacement rate treatments showed similar contribution rates. Insufficient nitrogen supply accelerates post-flowering redistribution, hastens leaf senescence, reduces photosynthetic capacity, and impairs grain filling (Shen et al., 2025). The treatment using only chemical fertilizer exhibited vigorous growth during the vegetative stage due to rapid nutrient uptake. However, after the silking stage, nitrogen losses through volatilization, leaching, and runoff (Simelane et al., 2024) led to reduced nutrient availability. Organic fertilizer, in contrast, promoted sustainable nutrient cycling, reduced runoff, and enhanced soil carbon sequestration (Verma et al., 2020). Higher leaf nitrogen levels enhance photosynthesis, delay leaf senescence, and improve nitrogen metabolism (Zhou and Yang, 2023). However, fully replacing chemical fertilizers with organic fertilizers resulted in lower dry matter accumulation during the vegetative growth stage compared to treatments with full chemical fertilizer application or combined organic-chemical fertilizer application. This results in insufficient early crop development, reducing the green leaf area available for photosynthesis (LAI). Although photosynthetic capacity increases in organic fertilizer treatments after the R1 stage, delaying leaf senescence and extending maize grain filling time, it cannot fully compensate for the reduction in final yield.

### 4.5 Differences in yield and its constituent factors

Yield differences are often determined by the number of ears, grains per ear, and 100-kernel weight. Analysis in this study revealed that when ear numbers were comparable, the number of grains per ear was lowest in the treatment where organic fertilizer completely replaced chemical fertilizer (T1), while 100-kernel weight was lowest in the treatment where only chemical fertilizer was applied (CK). Previous research suggests that under low nitrogen conditions, the number of spikelets differentiated in the first ear of maize is reduced by 25%, and ear development is delayed relative to tassel development, the relative elongation rate of silks is reduced, and asynchronous flowering increases (Jacobs and Pearson, 1991). The nutrient release rate of organic fertilizer alone is relatively slow, failing to meet crop nutrient demands throughout the entire growth period. This can easily lead to nutrient deficiencies in crops, affecting final yield. Differences in 100-kernel weight are often caused by variations in the grain filling period. The decline in fertilizer utilization efficiency in the later stages of the treatment with full chemical fertilizer application accelerates leaf senescence, shortening the grain filling period and resulting in reduced 100-kernel weight. Combined application of organic and chemical fertilizers avoids these issues. Partial substitution of chemical fertilizers with organic fertilizers maintains high productivity while improving soil conditions (Bai et al., 2018; Côté and Khiari, 2026). Nitrogen-reduced treatments supplemented with organic fertilizer increased post-flowering dry matter accumulation, post-flowering transport rates and contribution rates, and improved kernel weight (Ouyang et al., 2021).

Correlation analysis of yield components and parameters related to late-stage material accumulation revealed that dry matter accumulation and grain number per panicle are key factors influencing crop yield. Previous studies generally suggest that the substitution rate of organic fertilizer should not exceed 50%, with significant rice yield increases of 5.9% only observed when the substitution rate is below 60% (Gao et al., 2015; Yu et al., 2021; Li et al., 2025a). When the organic fertilizer substitution rate exceeds 60%, yields show no significant change, and the applied chemical fertilizers are not fully absorbed and utilized by crops, with the actual nitrogen fertilizer utilization efficiency being approximately 42% (Yu et al., 2022). Numerous studies have shown that the combined application ratio of organic fertilizer to chemical fertilizer often falls between 10% and 20%. One study found that compared with synthetic fertilizer alone, 20% organic substitution increased photosynthesis by 5.6% and maize yield by 7.9% (Han et al., 2025). Another study found that after three consecutive years of replacing chemical fertilizer with organic fertilizer, the treatment with 24% organic substitution achieved better effects than other substitution ratios and was significantly superior to conventional chemical fertilizer (Lu et al., 2024). Another study showed that an organic substitution ratio of 12.5% increased maize yield by 6.60% and biomass by 4.59% (Chen et al., 2025). These results are similar to our findings.

This study found that final yields were highest in the treatment combining organic and chemical fertilizers, followed by the treatment using only chemical fertilizers, and then the treatment using only organic fertilizers. Among the organic-chemical fertilizer combinations, the treatment substituting 10% of chemical fertilizer with organic fertilizer yielded the highest results in 2024, while substituting 20% of chemical fertilizer with organic fertilizer proved optimal in 2025. Comparing climatic conditions across the two years, average daily sunshine hours in July and August 2025 exceeded those of 2024. Since the silking and grain filling stages of spring maize in Northeast China typically occur during July and August, these months constitute a critical reproductive growth period for maize in the region. The study indicates that reduced solar radiation significantly lowers crop yields across all growth stages, particularly during grain filling (Shao et al., 2021). Reduced solar radiation after heading leads to decreased maize yield and biomass (Sun et al., 2023), and reducing light during 15–28 days after pollination inhibits the development of grain starch granules and the grain-filling process (Li et al., 2024), which is consistent with our findings. The leaf senescence process and the active grain-filling period of grains in 2025 were both higher than those in 2024, and the longer day length increased the grain-filling duration, which allowed the treatments with complete application of organic fertilizer to utilize soil nutrients more fully, showing that the maize yield in 2025 was significantly higher than that in 2024, reducing the differences between it and other treatments. Meanwhile, the yields of the three treatments with combined application of organic and chemical fertilizers also showed a clear increasing trend with higher amounts of combined organic fertilizer. Overall, setting the replacement ratio of organic fertilizer to chemical fertilizer in the range of 10%-20% is the most effective for increasing and stabilizing yield.

## 5 Conclusion

The combined application of organic and chemical fertilizers optimizes the carbon-nitrogen metabolic balance in leaves, enhances photosynthetic performance and delays the senescence process, effectively prolongs the effective grain-filling period of grains, thereby promoting dry matter accumulation and yield formation. Among them, replacing 10% of chemical fertilizer with organic fertilizer shows the best effect on rapid nutrient accumulation during the early stage of maize, while the treatment with 20% organic fertilizer replacement better balances nutrient supply during the late growth stage. Overall, replacing 10%-20% of chemical fertilizer with organic fertilizer achieves the best yield-increasing effect, and the combined application of organic and chemical fertilizers simultaneously improves yield and nutrient use efficiency.

## Data Availability

The original contributions presented in the study are included in the article/**Supplementary Material**, further inquiries can be directed to the corresponding author.

## Supplementary material

Supplementary Figure 1 Meteorological conditions during the maize growing season at the experimental site.
